# Disentangling structural genomic and behavioural barriers in a sea of connectivity

**DOI:** 10.1111/mec.15010

**Published:** 2019-03-15

**Authors:** Julia M. I. Barth, David Villegas‐Ríos, Carla Freitas, Even Moland, Bastiaan Star, Carl André, Halvor Knutsen, Ian Bradbury, Jan Dierking, Christoph Petereit, David Righton, Julian Metcalfe, Kjetill S. Jakobsen, Esben M. Olsen, Sissel Jentoft

**Affiliations:** ^1^ Centre for Ecological and Evolutionary Synthesis (CEES) Department of Biosciences University of Oslo Oslo Norway; ^2^ Zoological Institute University of Basel Basel Switzerland; ^3^ Department of Ecology and Marine Resources Mediterranean Institute for Advanced Studies, IMEDEA CSIC-UIB Esporles Spain; ^4^ Department of Ecology and Marine Resources Institute of Marine Research, (IIM CSIC) Vigo Spain; ^5^ Institute for Marine Research Flødevigen Norway; ^6^ Centre for Coastal Research University of Agder Agder Norway; ^7^ Oceanic Observatory of Madeira Funchal Portugal; ^8^ Department of Marine Sciences ‐ Tjärnö University of Gothenburg Gothenburg Sweden; ^9^ Science Branch, Fisheries and Oceans Canada St. John's Newfoundland and Labrador Canada; ^10^ GEOMAR Helmholtz Centre for Ocean Research Kiel Germany; ^11^ Centre for Environment, Fisheries and Aquaculture Science (CEFAS) Lowestoft UK

**Keywords:** adaptation, Atlantic cod, behavioural traits, chromosomal rearrangements, gene flow, sympatric divergence

## Abstract

Genetic divergence among populations arises through natural selection or drift and is counteracted by connectivity and gene flow. In sympatric populations, isolating mechanisms are thus needed to limit the homogenizing effects of gene flow to allow for adaptation and speciation. Chromosomal inversions act as an important mechanism maintaining isolating barriers, yet their role in sympatric populations and divergence with gene flow is not entirely understood. Here, we revisit the question of whether inversions play a role in the divergence of connected populations of the marine fish Atlantic cod (*Gadus morhua*), by exploring a unique data set combining whole‐genome sequencing data and behavioural data obtained with acoustic telemetry. Within a confined fjord environment, we find three genetically differentiated Atlantic cod types belonging to the oceanic North Sea population, the western Baltic population and a local fjord‐type cod. Continuous behavioural tracking over 4 year revealed temporally stable sympatry of these types within the fjord. Despite overall weak genetic differentiation consistent with high levels of gene flow, we detected significant frequency shifts of three previously identified inversions, indicating an adaptive barrier to gene flow. In addition, behavioural data indicated that North Sea cod and individuals homozygous for the LG12 inversion had lower fitness in the fjord environment. However, North Sea and fjord‐type cod also occupy different depths, possibly contributing to prezygotic reproductive isolation and representing a behavioural barrier to gene flow. Our results provide the first insights into a complex interplay of genomic and behavioural isolating barriers in Atlantic cod and establish a new model system towards an understanding of the role of genomic structural variants in adaptation and diversification.

## 1 INTRODUCTION

How new species arise and adapt to their environments is a fundamental question in the field of evolutionary biology. Yet, our understanding of the genetic mechanisms behind speciation with gene flow is far from complete (Jorde, Andersson, Ryman, & Laikre, 2018; Ravinet et al., 2017). Within the last decade, it has become accepted that population divergence, adaptation, and speciation in the face of gene flow is no rare exception (Hey, 2006; Nosil, 2008), and advances in sequencing technology have begun to reveal the underlying genomic architecture of this complex process (Seehausen et al., 2014; Tigano & Friesen, 2016; Wellenreuther & Bernatchez, 2018; Wolf & Ellegren, 2017). Several studies recently highlighted the role of genomic structural variants such as inversions in adaptation and diversification (e.g., Fishman, Stathos, Beardsley, Williams, & Hill, 2013; Jones et al., 2012; Kirkpatrick & Barton, 2006; Lohse, Clarke, Ritchie, & Etges, 2015; Lowry & Willis, 2010). Such rearranged regions may constitute intrinsic postzygotic barriers to gene flow (through genetic incompatibilities), extrinsic postzygotic barriers (where hybrids suffer reduced fitness in the parental environment), or act through ecological adaptive barriers where sets of locally adaptive alleles are captured and protected against recombination, giving a selective advantage to the individuals carrying the rearranged regions and leading to their spread in the respective environment (Feder, Gejji, Yeaman, & Nosil, 2012; Kirkpatrick & Barton, 2006; Rieseberg, 2001). The presence of structural rearrangements can therefore promote early stages of ecological divergence, which may eventually lead to speciation (Feder, Nosil, Wacholder, et al., 2014). Although theory predicts that in the face of gene flow, few large‐effect alleles may similarly spread and contribute to divergence if selection is strong enough, adaptive alleles rather persist if architecturally linked (Yeaman & Otto, 2011; Yeaman & Whitlock, 2011). Indeed, comparisons of sister species of rodents and birds revealed that sympatric sister species are more likely to differ by chromosomal rearrangements than allopatric ones (Castiglia, 2013; Hooper & Price, 2017).

In the marine fish Atlantic cod (*Gadus morhua*), four large (5–17 Mb) chromosomal rearrangements, each possessing high internal linkage disequilibrium (LD), have been detected (Berg et al., 2016; Kirubakaran et al., 2016; Sodeland et al., 2016), which show clinal distributions amongst the majority of populations throughout the entire species range in the North Atlantic Ocean, indicating a role in adaptation to local environments (Barth, Berg, et al., 2017; Berg et al., 2016, 2017; Kirubakaran et al., 2016; Sodeland et al., 2016; Star et al., 2017). It is likely that all of these rearrangements represent old chromosomal inversions (~2 million years based on divergence time estimates of closely related species (Matschiner et al., 2015; Kirubakaran et al., 2016), hereafter for simplicity termed inversions), which may have evolved in allopatric refugia during Pleistocene glaciation cycles (Kirubakaran et al., 2016). As a marine batch spawner without any brood care, releasing thousands to millions of pelagic eggs to be distributed by ocean currents, Atlantic cod reside in environments that a priori seem to provide little boundaries to restrict connectivity amongst populations (Hutchings, Bishop, & McGregor‐Shaw, 1999; Munk, Larsson, Danielssen, & Moksness, 1999; Cowen & Sponaugle, 2009; but see also Espeland, Albretsen, Olsen, & Bodvin, 2015). The inverted regions have therefore been associated with ecological adaptation of Atlantic cod to temperature (Bradbury et al., 2010; Therkildsen et al., 2013), oxygen and salinity (Berg et al., 2015), coastal environments (Barth, Berg, et al., 2017; Sodeland et al., 2016), and migration behaviour (Berg et al., 2016; Hemmer‐Hansen et al., 2013; Karlsen et al., 2013; Kirubakaran et al., 2016; Sinclair‐Waters et al., 2018). Although in some populations one or several of the inverted arrangements have reached near fixation, others represent strong frequency shifts relative to the ancestral arrangements, yet others frequently possess both arrangements in similar numbers (Barth, Berg, et al., 2017; Berg et al., 2016, 2017; Kirubakaran et al., 2016; Sodeland et al., 2016; Star et al., 2017).

In addition to structural rearrangements of the genome acting as postzygotic barriers to restrict gene flow between sympatric populations, prezygotic mechanisms including fitness advantages in the local environment, temporal, spatial or ecological shifts during the breeding season (causing allopatric reproduction), as well as behavioural differences (e.g., assortative mating) may also play a role (Coyne & Orr, 2004; Jones, Brown, Pemberton, & Braithwaite, 2006). For Atlantic cod, behavioural features that may contribute to prezygotic isolation such as spawning site fidelity, natal homing (Bonanomi et al., 2016; Espeland et al., 2007; Skjæraasen, Meager, Karlsen, Hutchings, & Fernö, 2011; Villegas‐Ríos, Réale, Freitas, Moland, & Olsen, 2017) and mate choice, as well as territorial behaviour (Hutchings et al., 1999), have been described, questioning the hitherto outlined prominent role of ecological adaptation through the chromosomal inversions in the ecotype divergence of this species.

To investigate the relative roles of different barrier mechanisms for the potential of adaptation and diversification in sympatric populations characterized by homogenizing gene flow, here we focus on a topographically restricted coastal fjord ecosystem in which the occurrence of three genetically differentiated types of Atlantic cod, a resident fjord‐type, an oceanic North Sea‐type and a western Baltic‐type, have been reported (Barth, Berg, et al., 2017; Knutsen et al., 2018). Sympatric populations that are connected and exposed to gene flow, but exhibit distinct phenotypic or behavioural differences, represent well‐suited systems to investigate genomic signatures of divergence because in these systems differentiated sites are expected to be rare and restricted to regions involved in adaptation (Coyne & Orr, 2004). High connectivity through geographic overlap between the different cod types has previously been demonstrated (André et al., 2016; Barth, Berg, et al., 2017), and—despite low overall genetic differentiation—significant shifts in frequency of the inverted arrangements have been observed (Barth, Berg, et al., 2017; Sodeland et al., 2016). However, whether the adaptive properties of the inversions constitute the only barrier to gene flow, or whether other barriers may also play a role, is not known. For example, yet undetected additional genomic structural variation in the form of large‐effect alleles (Yeaman & Otto, 2011), or physical barriers such as seascapes and salinity, oxygen, or temperature gradients (Ciannelli et al., 2010; Howe et al., 2010; Rogers, Olsen, Knutsen, & Stenseth, 2014), as well as behavioural differences could act as additional mechanisms restricting connectivity and gene flow.

Along the convoluted Norwegian coast, Atlantic cod show high site fidelity and restricted movement in studies using acoustic telemetry and longer‐term mark–recapture data (Aalvik, Moland, Olsen, & Stenseth, 2015; Freitas, Olsen, Knutsen, Albretsen, & Moland, 2016; Olsen & Moland, 2011; Rogers et al., 2014; Villegas‐Ríos, Moland, & Olsen, 2016; Villegas‐Ríos et al., 2017), limiting the potential for adult dispersal. However, long‐distance spawning migrations have also been recorded (Neat et al., 2014; Skjæraasen et al., 2011). Resident local behavioural units with spawning aggregations along the coast and inside the fjords, as well as physical retention of pelagic eggs through a fjord‐inward flow (Ciannelli et al., 2010), may thus explain the occurrence of different cod types and the genetic structuring documented in previous studies (Jorde, Knutsen, Espeland, & Stenseth, 2007; Knutsen et al., 2011). Yet, the degree to which settled juveniles stay and recruit to the local adult population is not known. Nevertheless, the relatively stable coexistence of early‐stage eggs, larvae and adults in the spawning condition of at least two distinct Atlantic cod types indicates that several populations use the convoluted coastline for spawning, as nursery, and for long‐term residence (Barth, Berg, et al., 2017; Jorde, Synnes, Espeland, Sodeland, & Knutsen, 2018; Knutsen et al., 2018; Rogers et al., 2014).

By using whole‐genome sequencing data of more than 200 Atlantic cod specimens from one fjord and six adjacent locations, we here gain comprehensive insights into the genomic variation and population relationships of the fjord cod community. To clarify how these highly connected cod types maintain differentiation, we characterize potential barriers to gene flow in the genomic landscape. Additionally, we test whether behavioural differences, potentially acting as prezygotic barriers to gene flow, are present among the cod types and whether these are correlated with the inversion states.

## 2 MATERIALS AND METHODS

### 2.1 Sampling and bioinformatics

**Figure 1:**
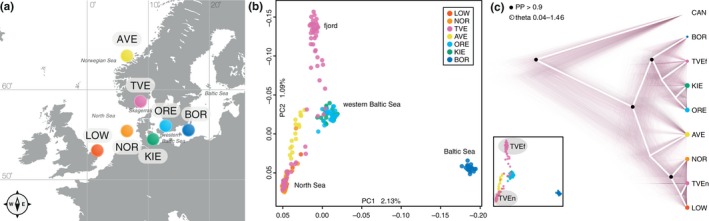
Genomic variation and population relationships. (a) Map of the study area with sampling sites depicted as coloured points: AVE: Averøya; LOW: Lowestoft; NOR: North Sea; TVE: Tvedestrand; ORE: Öresund; KIE: Kiel Bight; BOR: Bornholm. Colours match sampling sites in all figures. (b) Principal components analysis of genotypes excluding linkage groups LG01, 02, 07, and 12. Shown are the two main principal component axes (PC1, PC2); main clusters are identified as belonging to the North Sea, eastern Baltic Sea, western Baltic Sea and fjord‐type cod. (c) Phylogenetic relationships inferred using the multispecies coalescence model. Canadian individuals (CAN) were used to root the tree; the Tvedestrand sample was divided into North Sea‐type individuals (TVEn) and fjord‐type individuals (TVEf, see inset). Node support is shown as Bayesian posterior probabilities (PP), estimated population sizes are shown by point size (theta), the white line follows the maximum clade credibility summary tree, and thin coloured lines show a subsample of all inferred trees

A total of 204 individuals of Atlantic cod (*Gadus morhua*) from seven sites were sampled (Figure 1a, Supporting Information Table S1): Averøya, North Atlantic (AVE, *N* = 20), North Sea (LOW, *N* = 24; NOR, *N* = 24), Skagerrak Tvedestrand fjord (TVE, *N* = 70), and the western (ORE, *N* = 21; KIE, *N* = 22) and eastern Baltic Sea (BOR, *N* = 23). For all TVE individuals, both genomic and behavioural data were collected. DNA extraction, library preparation (Illumina Truseq DNA PCR‐free kit, combinatorial dual index adapters), and sequencing (Illumina HiSeq 2500, V4 chemistry, 2 × 125 bp paired‐end) was performed at the Norwegian Sequencing Centre according to the Centre's protocols (for a brief description see Barth, Damerau, Matschiner, Jentoft, & Hanel, 2017). Index‐hopping (Kircher, Sawyer, & Meyer, 2012) should not constitute a problem given the sequencing strategy used. Mapping and genotype calling were performed following the method described by Star et al. (2017), employing the paleomix version 1.5 (Schubert et al., 2014) pipeline for read processing and mapping (bwa mem version 0.7, Li & Durbin, 2009) against the GadMor2 genome (Star et al., 2011; Tørresen et al., 2017), and gatk haplotypecaller version 3.4.46 (McKenna et al., 2010) for variant calling. Average read depth per sample was 9.84 ± 1.16 (Supporting Information Table S1). Filtering was performed using bcftools version 1.3 (Li, 2011), vcftools version 0.1.14 (Danecek et al., 2011) and plink version 1.9 (Purcell et al., 2007) according to the following thresholds: FS < 60, MQRankSum > −12.5, ReadPosRankSum > −8, QD > 2, MQ > 40, SnpGap = 10, number of indels = 0, number of alleles = 2, meanDP < 30, GQ > 20, DP > 3, missing data <20%, minor allele count > 2, minor allele frequency (MAF) > 0.03, heterozygous excess (*p* < 0.001), and intrachromosomal LD (window size 10 kb; pairwise *r* ^2^ threshold 0.8). Additionally, repetitive regions were excluded (Tørresen et al., 2017), leading to a data set of 1,258,658 single nucleotide polymorphisms (SNPs). For population genomic and phylogenetic analyses, linkage groups (LGs) 01, 02, 07, and 12 were excluded due to the presence of large inversions (Berg et al., 2016; Kirubakaran et al., 2016; Sodeland et al., 2016). Phylogenetic analyses were performed without MAF filtering. Because of biased call probability of heterozygotes at low sequencing depth (Nielsen, Paul, Albrechtsen, & Song, 2011), which does not affect individual‐based analyses (Supporting Information Figure S1) but can influence the estimates of population genetic parameters due to an overall higher or lower coverage per population, analyses of the genomic landscape were performed using a data set in which genotypes with fewer than seven reads (DP < 7) for a sample were set missing.

### 2.2 Population differentiation and phylogenetic analyses

Genome‐wide population structure was inferred by performing hierarchical principal component analyses (PCA) using smartPCA in eigensoft version 6.0.1 (Patterson, Price, & Reich, 2006) including the function “lsqproject,” and through model‐based clustering using admixture version 1.3 (Alexander, Novembre, & Lange, 2009). Mean standard errors for admixture point estimates were calculated using 100 block‐bootstrap resamplings. Three replicates, each testing for one to six clusters (*k*) and 10‐fold cross‐validation was performed. Maximum likelihood phylogenetic inference was performed using raxml version 8.2.4 (Stamatakis, 2014) under the GTRCAT model with ascertainment bias correction and 100 bootstrap replicates on a data set including five randomly selected individuals per population. Only variable SNPs (781,038) were included and heterozygous SNPs were translated to ambiguity codes. The sister species of the Atlantic cod, the Alaska pollock (*Gadus chalcogrammus*; Malmstrøm et al., 2016), was used to root the tree. Homologous sites of *G. chalcogrammus* were identified through mapping against GadMor2 as described above and through retrieving the consensus sequence using “mpileup” in samtools version 1.3 (Li, 2011) for sites with MQ > 40. Five Canadian Atlantic cod individuals were included for subsequent rooting of the snapp phylogeny (see below). The species tree and population sizes were estimated using the multispecies coalescence approach in the add‐on snapp version 1.3.0 (Bryant, Bouckaert, Felsenstein, Rosenberg, & RoyChoudhury, 2012) for beast2 v2.4.7 (Bouckaert et al., 2014), using the same individuals as for the maximum likelihood analysis (except *G. chalcogrammus*), but allowing no missing data. As snapp runtimes are very long on larger SNP sets, the data set was reduced by applying a minimum distance of 70,000 bp between SNPs, resulting in 3,307 SNPs. The script “snapp_prep.rb” (Stange, Sánchez‐Villagra, Salzburger, & Matschiner, 2018) was used to prepare the input XML retaining the original settings except that theta values were not linked among branches. Three replicate analyses with random starting trees and a length of 1 million Markov chain Monte Carlo (MCMC) steps were carried out. Convergence was assessed by effective sample size (ESS) values > 200 (Rambaut, Drummond, Xie, Baele, & Suchard, 2018) after discarding the first 5% of each chain as burnin and merging the posterior distributions. The summary tree cloudogram (see Figure 1c) represents the entire tree set (28,503 trees), while for the representation of topology uncertainties only every 1,000th tree was sampled.

### 2.3 Genomic landscape

To identify structural genomic barriers to gene flow, we used an equal number of 20 individuals (randomly downsampled) from each of the identified co‐occurring North Sea (TVEn), western Baltic (KIE) and fjord‐type (TVEf) populations (where TVEn and TVEf are the TVE specimens showing the North Sea and the fjord genotype, respectively, see Section 3.1; KIE was chosen as the representative population for the western Baltic because only three western Baltic‐type cod were collected in the fjord, see Figure 1b). Nonoverlapping 50‐ and 100‐kb windowed chromosome scans to calculate the following measurements were performed using custom scripts: the pairwise fixation index (*F*_ST_; as in Weir & Cockerham, 1984), the pairwise between‐population sequence divergence (*d*_xy_; as in Ruegg, Anderson, Boone, Pouls, & Smith, 2014), the proportion of fixed differences (*d*_f_; see Ruegg et al., 2014) and the nucleotide diversity (π). To detect divergent regions <50 kb, we additionally ran bayescan version 2.1 (Foll & Gaggiotti, 2008) for each LG, evaluating the population pairs TVEf–TVEn, TVEf–KIE and TVEn–KIE using default settings, but adjusting the prior odds (PO) for the neutral model to 1,000 due to the large number of SNPs. Convergence was assessed by ESS > 200 using the coda package version 0.18 (Plummer, Best, Cowles, & Vines, 2006) in r version 3.3.3 (R Core Team 2018). Genes and their associated Gene Ontology (GO) terms in diverged regions (for the inverted regions on LG02, 07 and 12 see Barney, Munkholm, Walt, & Palumbi, 2017) were identified based on the GadMor2 genome annotation (Tørresen et al., 2017), considering all SNPs detected as outliers in the bayescan analysis (log_10_(PO) > 1, false discovery rate [FDR] 0.05) as well as genes within 5 kb of such outlier SNPs. Enrichment tests for GO terms were performed for genes detected in comparisons TVEf–TVEn and TVEf–KIE, but not TVEn–KIE using Fisher's exact test with the algorithm “weight01” in the topgo package version 2.26 (Alexa & Rahnenfuhrer, 2016) for r. All genes located in the highly differentiated region on LG16 (see Results) were included in the GO‐term test. The squared correlation between SNPs as a measure of LD (*r* ^2^; using plink) was calculated using all 204 individuals. SNPs in regions of putative inversions (LG01: positions 9,114,741–26,192,386; LG02: positions 18,609,260–23,660,985; LG07: positions 13,622,710–23,019,113; LG12 positions: 426,531–13,445,150; see Barth, Berg, et al., 2017; Berg et al., 2016) were extracted, and the inversion state of all specimens was analysed for each of these regions using smartPCA. Bootstrapping (Efron, 1979; sample size 1,000,000) of individual genotypes was used to assess a possible overrepresentation of the inverted arrangement (or the ancestral arrangement, referring to the overall frequency among all samples) of all inverted regions, under the null hypothesis that the frequency of the tested arrangement within a population corresponds to its overall frequency across all populations. Bonferroni correction was applied in r to correct for multiple comparisons (Rice, 1988). Maximum likelihood phylogenetic inferences were performed using raxml as described above, including only sequences of individuals homozygous for either the respective ancestral or the inverted arrangement.

### 2.4 Behavioural analyses

A total of 70 Atlantic cod (mean body length 46 cm, range 30–75 cm) were captured and tagged in the Tvedestrand fjord in May 2011–2013 as described elsewhere (Olsen, Heupel, Simpfendorfer, & Moland, 2012). Briefly, fish were captured using fyke‐net fishing for 1–3 days. Fish selected for tagging were anesthetized, equipped with an acoustic transmitter (Vemco V9P‐2L, 508–660 days battery life), programmed to send the current depth and a fish identification code (fish ID) every 110–250 s. Transmitters recorded on average 16,402 (±5,489) locations per fish, in 2011 and 2012 a maximal depth of 100 m (0.44‐m resolution and 5‐m accuracy), and in 2013 a maximum depth of 50 m (0.22‐m resolution and 2.5‐m accuracy). A fin‐clip was taken and stored in ethanol for DNA analysis. Fish were released at their initial capture location. Previous experiments showed no mortality in the tagging process (Olsen & Moland, 2011; Olsen et al., 2012). An array of 33 underwater ultrasonic receivers (Vemco VR2W, 69 kHz) was used to record and log transmitter signals (Villegas‐Ríos et al., 2017).

For each tagged fish, centres of activity (COAs) were calculated in 30‐min time bins following Simpfendorfer, Heupel, and Hueter (2002). Depth data time series and COA latitude/longitude plots were used to identify and remove all detections recorded after cessation of movement, i.e., death of the fish (Harrison et al., 2015). Code collisions and false detections were eliminated using a minimum of two detections per 24‐hr period. Diel vertical migration was estimated as the difference between average depth during the day and average depth at night, averaged over months (Freitas, Olsen, Moland, Ciannelli, & Knutsen, 2015). Monthly home range was estimated as the kernel utilization distribution with a probability level of 95% using all COAs from that month (fish were required to be present in the array during at least 20 days in any particular month). Monthly mean depth during daytime was calculated by averaging daily daytime mean depths.

Individual fate was assigned based on detection patterns. Fish were classified as either: (a) alive within the study area (i.e., multiple detections indicated horizontal and vertical movements), (b) dispersed from the study area (i.e., directional movement towards the outermost receivers followed by an absence of detections for the rest of the study) or (c) dead within the study area (i.e., when the fish either stopped transmitting while inside the study area or started transmitting continuously at the same depth).

Linear mixed effects models were used to analyse variation in cod behavioural traits (*BT*). The study area (a no‐take marine reserve) holds a key spawning locality for local cod (Ciannelli et al., 2010) and we specifically explored whether Atlantic cod displayed contrasting behaviour during the spawning season (January–April) compared to the feeding season (Espeland et al., 2007; Roney, Oomen, Knutsen, Olsen, & Hutchings, 2018). The full linear mixed effects model, prior to model selection, included fixed effects of season (*S*), inversion state for the LG02, LG07, and LG12 inverted regions (*LG02*,* LG07*,* LG12*), body size (*L*), and genetically determined sex (*GS*; Star et al., 2016). To explore our working hypothesis, we also included an interaction effect between season and inversion state:

$$\begin{array}{cc} \text{BT}= & c_{0}+c_{1},S+c_{2},LG02+c_{3},LG07+c_{4},LG12+c_{5}L+c_{6},GS+c_{7},S\times LG02+\\ & c_{8},S\times LG07+c_{9},S\times LG12+c_{10},S\times L+c_{11},S\times GS \end{array},$$

where *c*_0_ is the intercept. Season was modeled as a factor with two levels (spawning and feeding), cod inversion states as factors with three levels (ancestral, heterozygous and inverted), body size as a continuous variable, and genetic sex as a factor with two levels (female and male).

We included the following interaction effects: (a) between season and each of the inversions to explore whether any behavioural changes between feeding and spawning season depended on inversion state, (b) between season and body size because smaller fish might not be sexually mature and therefore not participate in spawning, and (c) between season and sex because spawning behaviour can differ between females and males, where males are known to defend territories associated with seafloor features during the spawning season (Meager et al., 2009, 2012). Three behavioural traits were analysed in separate models: (a) monthly home range size, (b) monthly mean depth use and (c) monthly mean diel vertical migration. Home range size was log‐transformed to stabilize the variance. Fish ID was included as a random effect to account for repeated (monthly) observations on fish behaviour (among‐individual variance).

Next, we used the Lande–Arnold linear regression approach (Lande & Arnold, 1983) to model fitness as relative longevity (days survived/mean days survived, *S*). Fish that dispersed permanently from the study area were excluded from this analysis because their fate cannot be determined. The full model included effects of inversion states (*LG02*, *LG07* and *LG12*), body size (*L*) and genetic sex (*GS*):

$$S=c_{0}+c_{1},LG02+c_{2},LG07+c_{3},LG12+c_{4}L+c_{5},GS$$

In a final set of analyses, we replaced the inversion factor with a genotype factor where fish were defined as either North Sea‐type (TVEn) or fjord‐type (TVEf) based on genetic results (see Section 3.1). A total of 22 individuals could not be reliably defined as either of the two types (i.e., were classified as “intermediates”) and were excluded from these analyses.

We used the Akaike information criterion (AIC) for model selection (Burnham & Anderson, 1998). In a two‐step process, we first decided on the most parsimonious model structure for effects of body size and sex on behaviour, while maintaining all inversion state effects of interest. Next, we selected the most parsimonious model structure for inversion state effects on behaviour.

## 3 RESULTS

### 3.1 Genomic variation and population relationships

Genomic variation and relationships among all sampled specimens (Figure 1a) were explored using multivariate and clustering analyses, as well as phylogenetic methods. Applying PCA revealed the largest differentiation to be found between the eastern Baltic Sea (BOR) and all other samples, while the second largest variation was found between the combined North Sea cluster (NOR, LOW) and some of the fjord specimens (TVE) (Figure 1b). The western Baltic samples (ORE, KIE) formed a separate, intermediately placed cluster, while the Norwegian coastal individuals (AVE) were recovered between the North Sea, the western Baltic, and the fjord (TVE) cluster. Individuals collected in the fjord (TVE) showed a split distribution, with about half of the specimens (*N* = 21) grouping with the North Sea cluster, while the remaining ones (*N* = 27) formed a well‐defined private cluster (Figure 1b, fjord). Three TVE specimens were also placed within the western Baltic cluster, one within the Norwegian coastal cluster, and some individuals (*N* = 18) were distributed between the main clusters on PC2 and could not be clearly assigned to a particular cluster (referred to as “intermediate” in Section 3.3, see also Supporting Information Table S2). Hierarchical exclusion of the most differentiated clusters (BOR, NOR, LOW), as well as separate analyses of the TVE specimens and the North Sea cluster, respectively, confirmed this overall pattern of population differentiation (Supporting Information Figure S1b–f). Using the software admixture for maximum likelihood model‐based inference of genomic variation, the best‐supported models (lowest cross‐validation error) were obtained assuming one to three clusters. As in the PCA results, the admixture ancestry proportions yielded three main clusters represented by the eastern Baltic Sea sample, the North Sea samples and half of the TVE sample, while the ancestry proportions of the western Baltic and Norwegian coastal samples appeared admixed (Supporting Information Figure S2a–c).

To obtain estimates of between‐population relationships, we constructed a phylogeny using the multispecies coalescence approach. Because the population genomic analyses above suggested the existence of distinct genotype units within the TVE sample, we subdivided the TVE specimens into a “fjord‐type” termed TVEf and a North Sea‐type termed TVEn according to their position in the PCA (Figure 1c inset, Supporting Information Table S2). As a closely related outgroup, we chose five Atlantic cod specimens from the western North Atlantic (Twillingate, Canada; CAN), which were identified as a sister population to our eastern North Atlantic samples based on a maximum likelihood phylogeny rooted using the sister species of Atlantic cod, the Alaska pollock (Supporting Information Figure S3). The summary tree (Figure 1c) shows two well‐supported monophyletic clades: one for the combined group of samples from the eastern Baltic Sea (BOR) and the western Baltic Sea (ORE, KIE), which also included the fjord‐type TVE specimens (TVEf), and another for the North Sea populations (NOR, LOW), which included the North Sea‐type TVE individuals (TVEn). Relative divergence times indicate that the eastern Baltic Sea population is evolutionarily older than the TVEf and the western Baltic populations. Relative effective population sizes are larger in the North Sea (θ = 1.14) and western Baltic (θ = 1.43, KIE; and 1.34, ORE) than in the eastern Baltic Sea (θ = 0.04) and within the TVEf cod (θ = 0.52; Figure 1c). Alternative topologies indicate topological uncertainty due to incomplete lineage sorting or existing gene flow between the North Sea populations and the Baltic populations (Figure 1c).

### 3.2 Barriers to gene flow in the genomic landscape

**Figure 2:**
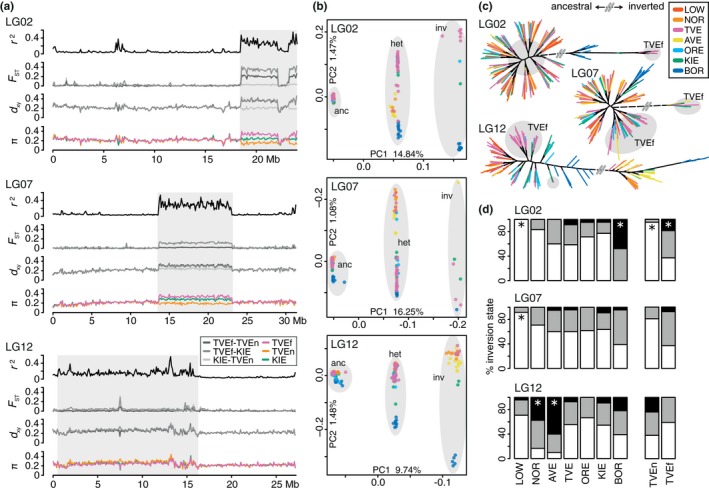
Barriers to gene flow in the genomic landscape. (a) Windowed measurements of linkage disequilibrium (LD, genotype correlation, *r* ^2^), pairwise population divergence (fixation index, *F*_ST_), pairwise between population sequence divergence (*d*_xy_) and nucleotide diversity (π) for linkage groups LG02, 07, and 12. Grey boxes outline the regions of large inversions. (b) Principal components analyses of variants within the inversions. Shown are the two main axes (PC1, PC2); main clusters are outlined by grey circles depicting individuals homozygous for the ancestral arrangement (anc), heterozygous (het) or homozygous for the inverted arrangement (inv). (c) Maximum likelihood trees of homozygous sequences within the inversions. The grey outline indicates placement of fjord‐type (TVEf) specimens. The grey double bar signifies discontinuation of the branch connecting ancestral (left) and inverted (right) arrangements. (d) Frequency of homozygous ancestral (white), homozygous inverted (black), and heterozygous (grey) individuals per sampling site, as well as within fjord‐type (TVEf) and North Sea‐type (TVEn) fish. * significantly overrepresented ancestral or inverted arrangements. Colours match sampling sites in Figure 1

Our population genomic analyses described the spatial and temporal co‐occurrence of three genetically differentiated types of Atlantic cod within the Tvedestrand fjord ecosystem (TVE North‐Sea type [TVEn], TVE fjord‐type [TVEf], and TVE western Baltic‐type cod). To identify genomic regions that may act as structural barriers to restrict gene flow between these groups, we performed genome scans testing for LD and differentiation. We identified three previously described large regions of strong LD and high interpopulation divergence on LG 02, 07, and 12 (Figure 2a, see Supporting Information Figure S4 for all chromosomes and measurements). Besides these three regions, no other large regions of elevated LD accompanied by genetic divergence indicating chromosomal rearrangements were detected. However, several genome‐wide distributed differentiation peaks, including a 117‐kb region on LG16 (15,467,682–15,584,985) showing no signs of strong LD, but consistently high *F*_ST_ (> 0.6, Supporting Information Figure S5), indicate the existence of further smaller regions of fjord‐type divergence. Fixed SNPs, commonly observed between diverging species, were not detected among the TVEn, TVEf and KIE individuals. As regions of tightly linked loci have been proposed as promoters of divergence in sympatric populations (Feder, Egan, & Nosil, 2012), SNPs within the three high LD regions were extracted and the inversion state for all regions, specimens, and populations, as well as their evolutionary relationships were analysed. Applying PCA segregated specimens into three clusters on the first principal component axis (Figure 2b), visualizing the bi‐allelic nature of the inversions in which homozygous individuals occurred in two groups, with heterozygous individuals located as intermediate, irrespective of sampling site. On the second axis, the ancestral arrangement showed relatively little divergence between populations (except for the eastern Baltic specimens on LG07), whereas higher divergence was observed between individuals carrying the inverted arrangement. Similar to the whole‐genome analysis, the arrangements of TVE individuals clustered with either North Sea or western Baltic individuals, as well as within a private cluster for the inversion on LG02. A phylogenetic approach revealed that these TVE individuals form a monophyletic group, which was well separated from the eastern Baltic individuals (Figure 2c). All of these TVE individuals belong to the fjord‐type (TVEf). Similarly, TVEf individuals were mostly recovered within shared or neighbouring clades for the ancestral and inverted arrangements of the inverted regions on LG07 and LG12. In contrast, TVEf individuals were recovered well dispersed among all other individuals sharing the ancestral LG02 arrangement (Figure 2c).

Frequency comparisons of the ancestral and inverted arrangements among populations revealed a significant overrepresentation of the ancestral LG02 arrangement in LOW (*p* < 0.001) as well as in TVEn (*p* < 0.01), and an overrepresentation of the inverted LG02 arrangement in BOR (*p* < 0.001) as well as in TVEf (*p* < 0.01) (Figure 2d, Supporting Information Table S3). On LG07, the ancestral arrangement was also found to be overrepresented in LOW (*p* < 0.01), whereas the inverted arrangement on LG12 was overrepresented in NOR (*p* < 0.01) and AVE (*p* < 0.001). For TVEf, we also observed an overrepresentation of the inverted LG07 (*p* = 0.0043) and the ancestral LG12 arrangement (*p* = 0.0041); however, both comparisons were not significant after correction for multiple comparisons. No significant yearly difference was detected within the TVE sample (Supporting Information Figure S6), and none of the inverted regions deviated from Hardy–Weinberg equilibrium (HWE) (*p* > 0.05; in LOW the inverted LG02 arrangement is not present). All of the inverted regions segregated independently, and all but three of the 27 possible combinations of homozygous ancestral, heterozygous and homozygous inverted arrangements on the three LGs were present. The nonsampled combinations are: (a) homozygous for all inverted arrangements, (b) homozygous for the inverted arrangement on LG02 and LG07 but heterozygous on LG12, and (c) heterozygous on LG02 but homozygous for the inverted arrangement on LG07 and 12. However, due to few inverted arrangements, the chance of sampling all combinations within 204 individuals is small (< 0.00001, based on the observed frequencies at each inverted region).

Several genome‐wide distributed differentiation peaks not showing high LD were identified (Supporting Information Figure S4), which may indicate genes under positive selection among the cod types. Bayesian analyses to detect candidate loci under positive selection identified significant SNPs (*q* < 0.05) between TVEf–TVEn and TVEf–KIE, excluding SNPs also detected in TVEn–KIE, in 20 genes on four LGs (Supporting Information Table S4). The GadMor2 genome assembly includes 14,060 predicted genes associated with GO terms (Tørresen et al., 2017), of which 13,977 had called SNPs within a region of 5,000 bp up‐ or downstream from the genes' coding sequence. GO enrichment analyses of the detected genes identified 12 significantly enriched GO terms (*p* < 0.05); however, none of these remained significant after FDR correction (Supporting Information Table S5).

### 3.3 Behavioural barriers to gene flow

To investigate whether possessing the inverted arrangements or genetically belonging to the fjord‐type (TVEf) was correlated with behavioural differences constituting an additional prezygotic barrier to gene flow, we used data from acoustically tagged specimens to calculate home range, mean depth, diel vertical migration, and survival.

#### 3.3.1 Home range

**Figure 3:**
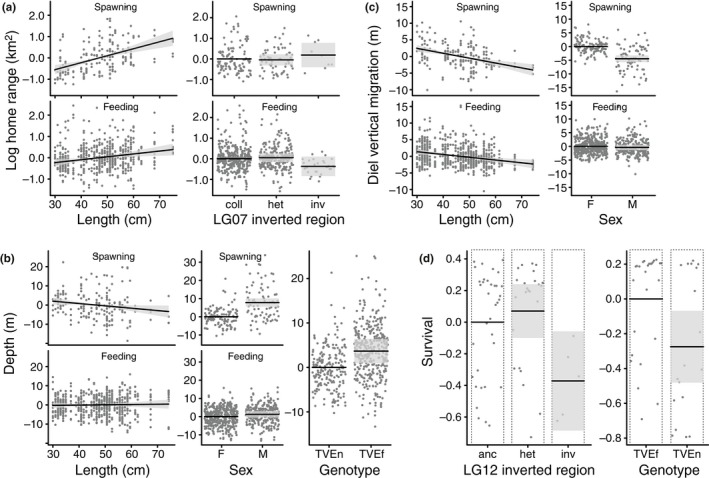
Survival and behavioural barriers to gene flow. Mean predictions (black lines) from models describing cod behaviour (a–c) and survival (d), also showing the 95% confidence bands (grey fields) and partial residuals (dots). For each model, the predictions are scaled against a reference level (zero). Survival is estimated as relative longevity (days survived/mean days survived). anc: ancestral; F: female; het: heterozygous; inv: inverted; M: male; TVEf: fjord‐type; TVEn: North Sea‐type

A mixed effects model supported two‐way interaction effects between the LG07 inversion state and season on cod home range size (Table 1, Figure 3a, see Supporting Information Figure S7 for individual raw values). A simplified model excluding the LG07 inversion factor, as well as a more complex model including an effect of the LG02 inversion state had similar support (Table 1). Specifically, all inversion states and body sizes tended to increase their home range during the spawning season compared to the feeding season, while this effect was stronger for fish having the inverted LG07 arrangement and for larger fish (Table 2, Figure 3a). A total of 25% of the variation in home range was associated with fish ID (among‐individual variance), while the fixed effects explained 18% of the variance. Alternative models, in which the inversion state was replaced with a factor describing genotype (TVEf vs. TVEn), revealed no significant effects of genotype, either as part of a two‐way interaction with season or as a simple additive effect on home range (*p* > 0.50).

**Table 1:** Comparison of linear mixed models for predicting Atlantic cod behaviour (home range, mean depth, and diel vertical migration), showing the structure and Akaike information criterion (AIC) of each model. Inversion state (*LG02*,* LG07* and *LG12*), season (*S*), and genetically determined sex (*GS*) were included as factors, while body length (*L*) was included as a linear covariate. Fish ID was included as a random effect (not shown)

Home range (HR) model	AIC
* HR = S′LG02 + S′LG07 + S′LG12 + S′L + S′GS*	1,689.3
* HR = S′LG02 + S′LG07 + S′LG12 + S′L + GS*	1,687.3
* HR = S′LG02 + S′LG07 + S′LG12 + S′L*	1,685.7
* HR = S′LG02 + S′LG07 + S′LG12 + L*	1,695.0
* HR = S′LG02 + S′LG07 + LG12 + S′L*	1,683.4
* HR = S′LG02 + S′LG07 + S′L*	1,679.9
* HR = S′LG02 + LG07 + S′L*	1,683.3
* HR = LG02 + S′LG07 + S′L*	1,677.8
* **HR** = **S′LG07 + S′L***	**1,677.0**
* HR = S′L*	1,677.3
**Mean depth (D) model**	
* D = S′LG02 + S′LG07 + S′LG12 + S′L + S′GS*	5,539.4
* D = S′LG02 + S′LG07 + S′LG12 + S′L + GS*	5,580.1
* D = S′LG02 + S′LG07 + S′LG12 + L + S′GS*	5,543.3
* D = S′LG02 + S′LG07 + LG12 + S′L + S′GS*	5,538.1
* D = S′LG02 + S′LG07 + S′L + S′GS*	5,537.3
* D = S′LG02 + LG07 + S′L + S′GS*	5,533.6
* D = S′LG02 + S′L + S′GS*	5,530.3
* D = LG02 + S′L + S′GS*	5,528.3
* **D = S′L** + **S′GS***	**5,525.5**
**Diel vertical migration (DVM) model**	
* DVM = S′LG02 + S′LG07 + S′LG12 + S′L + S′GS*	4,507.6
*DVM = S′LG02 + S′LG07 + S′LG12 + S′L + GS*	4,555.0
* DVM = S′LG02 + S′LG07 + S′LG12 + L + S′GS*	4,507.7
* DVM = S′LG02 + S′LG07 + LG12 + S′L + S′GS*	4,507.0
* DVM = S′LG02 + S′LG07 + S′L + S′GS*	4,505.9
* DVM = S′LG02 + LG07 + S′L + S′GS*	4,506.8
* DVM = LG02 + S′LG07 + S′L + S′GS*	4,506.4
* DVM = S′LG07 + S′L + S′GS*	4,503.3
* **DVM** = **S′L** + **S′GS***	**4,499.1**

Note

The most parsimonious model selected for inference is shown in bold.

**Table 2:** Parameter estimates (Par) with standard errors (*SE*) for the fixed effects included in the model selected for inference about variation in Atlantic cod behaviour. Cod having the ancestral chromosomal arrangement, the female sex and the feeding season were coded as zero in the model (reference levels)

Home range model	Par	*SE*	*p*‐Value
Intercept	−3.018	0.221	<0.001
LG07_het_	0.054	0.104	0.606
LG07_inv_	−0.368	0.238	0.125
Body length	0.014	0.005	0.006
Season_spawning_	−0.308	0.246	0.212
LG07_het_ × Season_spawning_	−0.098	0.112	0.383
LG07_inv_ × Season_spawning_	0.557	0.24	0.02
Body length × Season_spawning_	0.019	0.005	<0.001
**Mean depth model**			
Intercept	14.759	2.073	<0.001
Body length	0.015	0.043	0.735
Season_spawning_	3.694	2.343	0.115
Sex_male_	1.264	0.893	0.161
Body length × Season_spawning_	−0.135	0.047	0.004
Sex_male_ × Season_spawning_	6.541	0.969	<0.001
**Diel vertical migration model**			
Intercept	7.18	1.125	<0.001
Body length	−0.082	0.023	0.001
Season_spawning_	2.55	1.287	0.048
Sex_male_	−0.404	0.485	0.408
Body length × Season_spawning_	−0.063	0.026	0.016
Sex_male_ × Season_spawning_	−4.071	0.532	<0.001

Note

Het: heterozygous; inv: inverted.

#### 3.3.2 Mean depth during daytime

A mixed effects model supported two‐way interaction effects between body size and season, as well as sex and season on cod mean depth use during daytime (Table 1, Figure 3b). Alternative models including inversion state had little support (Table 1). Smaller cod tended to occupy deeper waters during the spawning season compared to larger cod (Table 2, Figure 3b). On average, males occupied deeper waters compared to females. During the spawning season, females shifted to somewhat shallower depths while this effect was the opposite for males (Table 2, Figure 3b). A total of 23% of the variation in mean depth use was associated with fish ID (among‐individual variance), while the fixed effects explained 9% of the variance. Alternative models, in which the inversion state was replaced with a factor describing genotype (TVEf vs. TVEn), revealed no significant interaction between genotype and season (*p* = 0.85), while there was statistical support for an additive effect of genotype where TVEf individuals tended to occupy deeper waters compared to the TVEn individuals (β_TVEf_ = 3.67, *SE* = 1.40, *p* = 0.012, fixed effects explained 12% of the variance), accounting for effects of body size and sex (Figure 3b).

#### 3.3.3 Diel vertical migration

As for mean depth, a mixed effects model supported two‐way interaction effects between body size and season, as well as sex and season, on cod diel vertical migrations (Table 1, Figure 3c). Alternative models including inversion states had little support (Table 1). On average, smaller fish had more extensive diel vertical migrations compared to larger fish and maintained this movement pattern during both feeding and spawning seasons (Table 2, Figure 3c). In contrast, larger fish only performed noticeable diel vertical migrations during the feeding season. On average, females displayed more extensive diel vertical migrations compared to males, especially during the spawning season where males showed signs of a reversed migration pattern (Table 2, Figure 3c). A total of 22% of the variation in diel vertical migration was associated with fish ID (among‐individual variance), while 18% was explained by fixed effects. Alternative models in which the inversion state was replaced by a factor describing genotype (TVEf vs. TVEn) revealed no significant effects of genotype, either as part of a two‐way interaction with season or as a simple additive effect on diel vertical migration (*p* > 0.48).

#### 3.3.4 Survival

A total of six fish permanently left the fjord for the outer coast during the 4‐year study period (*N*_North Sea_ = 3, *N*_fjord_ = 1, *N*_indermediate_ = 2). Of those fish that did not leave (*N*_dead_ = 32, *N*_alive_ = 32), a generalized linear model supported effects of the LG12 inversion state and body size on cod survival, although the support for an effect of body size was marginal (Table 3, Figure 3d). A total of 17% of the variation in survival was explained by the selected model. Alternative models including an effect of the LG02 and LG07 inversion states as well as sex received little support (Table 3). Predicted cod survival was lower for cod having the inverted LG12 arrangement and was also marginally lower for smaller fish compared to larger fish (Table 4). Alternative models, in which the inversion state was replaced with a factor describing genotype (TVEf vs. TVEn), predicted survival to be lower for the TVEn compared to the TVEf individuals (β_TVEn_ = −0.27, *SE* = 0.11, *p* = 0.013, Figure 3d). A total of 15% of the variation in survival was explained by the selected genotype model.

**Table 3:** Comparison of linear models for predicting Atlantic cod survival (*S*), showing the structure and Akaike information criterion (AIC) of each model. Inversion state (*LG02*,* LG07* and *LG12*) and genetically determined sex (*GS*) were included as factors, while body length (*L*) was included as a linear covariate

Cod survival model	AIC
*S = LG02 + LG07 + LG12 + L + GS*	51.4
*HR = LG02 + LG07 + LG12 + L*	51.9
*HR = LG02 + LG07 + LG12*	52.5
*HR = LG02 + LG12 + L*	47.9
***HR** = **LG12 + L***	**44.5**
*HR = LG12*	44.8
*HR = L*	47.7

Note

The most parsimonious model selected for inference is shown in bold.

**Table 4:** Parameter estimates (Par) with standard errors (*SE*) from the model selected for inference about variation in Atlantic cod survival. Individuals having the ancestral chromosomal arrangement and the female sex were coded as zero in the model (reference levels)

Cod survival	Par	*SE*	*p*‐Value
Intercept	0.702	0.211	0.002
LG12_het_	0.070	0.087	0.421
LG12_inv_	−0.372	0.160	0.024
Body length_._	0.007	0.004	0.140

Note

Het: heterozygous; inv: inverted.

## 4 DISCUSSION

What are the processes and mechanisms behind hampered gene flow in sympatric populations? Do inversions play a role in sympatric adaptation and diversification, and which other barriers may be important to reduce gene flow? These questions are central to our understanding of the mechanisms leading to speciation with gene flow. Using a unique data set combining genomic with behavioural data of highly connected wild Atlantic cod populations within a fjord ecosystem, we here show that significant frequency shifts of chromosomal inversions among cod types exist, one of which was also correlated with survival within the fjord environment, suggesting the existence of adaptive barriers to gene flow (i.e., a fitness effect associated with the environment). However, many weakly differentiated loci across the genome possibly under positive selection, as well as behavioural differences of depth usage between North Sea and fjord types suggest that further prezygotic barriers to gene flow may also exist.

### 4.1 Temporally stable sympatric occurrence of three genetically differentiated Atlantic cod types

Based on whole‐genome sequencing data (excluding the inversion‐carrying LGs), we demonstrated that the Atlantic cod community in the southern Norwegian fjord of Tvedestrand consists of three co‐occurring genetically differentiated cod types, of which two types are associated with the adjacent Atlantic cod populations in the North Sea and the western Baltic Sea, while the third forms a distinct unit that was only detected within the fjord (Figure 1b). Several observations suggest that this community structure is temporally stable. First, all three types were sampled from two consecutive years. Moreover, our behavioural data showed that only six out of the 70 tagged cod permanently left the fjord during their tracking period (~18 months) within the 4‐year study period, suggesting that the different cod types do not just visit the fjord occasionally during the mobile feeding season—as shown for Atlantic cod populations in the North Sea area (Neat et al., 2014)—but they are instead present in the fjord for extended periods of time. Furthermore, stable coexistence of juvenile North Sea and fjord‐type cod has been documented over a timescale of 14 years at several locations along the southern Norwegian coast (Knutsen et al., 2018). In addition, sympatric spawning is supported through the shared spawning period for North Sea and western Baltic cod from January to April (Hüssy, 2011; Neat et al., 2014), the occurrence of North Sea, western Baltic and fjord‐type adult cod in spawning condition in other southern Norwegian fjords (Barth, Berg, et al., 2017), and the simultaneous presence of young‐stage cod eggs in the fjords (Ciannelli et al., 2010; Jorde, Synnes, et al., 2018; Knutsen et al., 2007; Svedäng et al., 2018). Finally, male cod will typically defend territories at specific sites connected to the fjord bottom during the spawning period, and thus reduced diel vertical migration can be expected for individuals that take part in spawning (Meager et al., 2009, 2012). We detected reduced diel vertical migration during the spawning season for the fjord‐type cod (TVEf) as well as the North Sea‐type cod (TVEn), indicating that both types are active in spawning. All these observations of previous and the current study indicate a stable sympatric cod community within the Tvedestrand fjord. Although we found a very low abundance of the western Baltic‐type in the fjords, an earlier study in which several southern Norwegian fjords and a larger number of individuals were sampled detected almost equal numbers of North Sea, western Baltic and fjord‐type cod (Barth, Berg, et al., 2017).

Two questions then arise: Which barriers limit gene flow between sympatric cod types and maintain genetic differentiation? How does genetic exchange between North Sea and western Baltic‐type cod in the fjords with the respective offshore populations occur? Our phylogenetic relationships showed that the fjord (TVEf) and North Sea‐type (TVEn) appear in two different well‐supported clades, with TVEn nested among the North Sea populations with large topological uncertainties, underlining that these cod belong to the same genotypic unit (Figure 1c). The genetic similarity of TVEf and the western Baltic cod may further indicate an origin from the western Baltic population, which could have evolved as a response to random or nonrandom dispersal, driven, for example, by habitat matching due to similar habitats of reduced salinity (Edelaar & Bolnick, 2012). These data support a scenario of either recent (sympatric) divergence, or of allopatric divergence and secondary contact with (a) insufficient time for homogenization or with (b) reduced gene flow between fjord‐type (TVEf) with North Sea (TVEn) and western Baltic‐type cod. The latter is possibly due to the presence of reproductive barriers, while genetic exchange among oceanic populations is maintained through, for example, the regular supply of fjords with larvae of offshore origin, which has been documented to occur through oceanic drift of pelagic eggs and larvae (André et al., 2016; Barth, Berg, et al., 2017; Jonsson, Corell, André, Svedäng, & Moksnes, 2016; Stenseth et al., 2006). However, in this last scenario, whether the chromosomal inversions or other barriers maintain genetic separation remains uncertain.

### 4.2 Frequency shifts of inversions do not fully explain differentiation of Atlantic cod types

Chromosomal inversions can promote divergence through the suppression of recombination (Kirkpatrick & Barton, 2006), and numerous investigations in several organisms such as insects (Ayala, Guerrero, & Kirkpatrick, 2013; Lohse et al., 2015) and plants (Lowry & Willis, 2010; Twyford & Friedman, 2015), but also fish (Fan & Meyer, 2014; Jones et al., 2012; Kirubakaran et al., 2016) have described a central role of inversions in divergence with gene flow. This view is also supported by recent simulation studies (Feder, Nosil, & Flaxman, 2014; Yeaman, 2013). Genome scans of North Sea (TVEn), western Baltic (KIE) and fjord‐type (TVEf) cod revealed low genomic differentiation across most of the genome with the exception of three previously identified inverted regions on LG02, 07, and 12 (Figure 2a, Berg et al., 2016; Sodeland et al., 2016; Kirubakaran et al., 2016). Thus, these three inversions are prime candidates for a barrier mechanism that might act through either intrinsic (genome incompatibilities) or extrinsic (fitness effect associated with the environment) isolation.

Genetic characterization of the inversions revealed frequency shifts of the ancestral and inverted arrangements between TVEf and TVEn and bi‐allelic segregation, in which the ancestral arrangement shows less divergence among populations than the inverted arrangement (Figure 2b). Such bi‐allelic segregation can be attributed to diversifying selection acting on the inverted arrangement, or it could indicate that the ancestral, more common arrangement is subject to ongoing homogenization by gene flow, while recombination in the less frequent inverted arrangement is reduced. Interestingly, phylogenetic analyses revealed population‐specific clustering of the ancestral and inverted arrangements of fjord‐type cod (TVEf) on LG07 and 12, but not on LG02 (Figure 2c), suggesting that the inversions have independent properties, where the ancestral arrangement on LG02 experiences more exchange among populations than the ancestral arrangement of the other two LGs. Thus, sets of co‐adaptive alleles may be captured in one or several of the inverted regions and protected from recombination, creating an adaptive barrier to gene flow through the fitness advantage of individuals carrying the inversion in the local environment (Kirkpatrick & Barton, 2006). Indeed, for eastern Baltic cod living in low‐salinity conditions, key genes important for osmoregulation have been described in and around the inversion on LG02 (Berg et al., 2015), which could also provide adaptive value in fjord environments (Barth, Berg, et al., 2017). Furthermore, temperature adaptations through physiological adjustments and oxygen consumption (Grenchik, Donelson, & Munday, 2012) may be important properties in the fjord environment, which is characterized by stable and stratified temperatures and decreased oxygen concentrations in deeper layers (Halvorsen, 2013; Saetre, 2007). Notably, the inversions on LG02 and LG12 have been shown to contain genes associated with temperature and oxygen regulation (Berg et al., 2015; Bradbury et al., 2010; Therkildsen et al., 2013). However, GO enrichment analyses of genes within the inverted region on LG12 showed no significant enrichment, while in the inversion on LG02 genes involved in DNA/chromatin structuring were found to be significantly enriched, and the inversion on LG07 showed a significant enrichment of genes in signalling and metabolic processes (Barney et al., 2017). Adaptive genes residing in inversions have also been described in other species, for example the threespine stickleback (Jones et al., 2012) or the willow warbler (Lundberg et al., 2017). Yet, such correlations between inversions and the environment may also be caused by intrinsic genetic incompatibilities that merely coincide with ecological barriers (Bierne, Welch, Loire, Bonhomme, & David, 2011). In conflict with a hypothesized intrinsic postzygotic barrier is the fact that all possible 27 combinations of the three inverted regions have been observed in wild Atlantic cod (data not shown). In our data set, all but three of these 27 combinations occurred. However, due to the low frequency of inverted arrangements, the chance of sampling all combinations within ~200 individuals is small. On the other hand, an intrinsic postzygotic barrier where problems during meiotic chromosome pairing in heterozygotes may lead to sterility or underdominance cannot be ruled out, but seems unlikely given that none of the inversions was found to be fixed within a population, all rearrangement haplotypes conform to HWE expectations, and heterozygotes are abundant. In addition, extrinsic postzygotic barriers where hybrids are unfit in the local environment seem similarly unlikely, because we did not observe decreased fitness (survival) of heterozygotes for any of the inversions. However, significantly reduced fitness in the fjord environment of individuals homozygous for the LG12 inversion (Figure 3, Table 4) may indicate immigrant inviability (Nosil, Vines, & Funk, 2005) and selection against North Sea migrants, which were also shown to have an overrepresentation of the LG12 inverted arrangement (Figure 2d).

Although frequency shifts of the inverted arrangement indicate adaptive properties, and individuals homozygous for the LG12 inversion showed lower fitness in the fjord environment, the lack of fixed inverted arrangements implies that the inversions are not purely diagnostic for genotype fate. This is in contrast to the high degree of fixation of the LG01 inversion in Northeast Arctic cod, which has been linked to migratory behaviour (Berg et al., 2016; Kirubakaran et al., 2016). Behavioural traits, especially traits related to reproductive behaviour, have previously also been associated with inversions in the white‐throated sparrow (Tuttle et al., 2016) and the ruff (Küpper et al., 2016). However, in line with the lack of fixed inversions, support for a correlation between the inversions and the tested behavioural traits was weak and restricted to a tendency for larger home ranges during the spawning season in individuals having the LG07 inverted arrangement.

The lack of such a direct relationship between fjord‐type cod (TVEf) and the inverted arrangements suggests that adaptive alleles may perhaps have manifested elsewhere in the genome. In our genome scans, we did not detect other large regions (more than few kb) with tightly linked loci (indicating reduced recombination) that are also differentiated between TVEf and TVEn/KIE and thus may be protected from gene flow and have a barrier effect. However, it has been shown that chromosomal rearrangements are not always required to maintain a barrier to gene flow (Davey et al., 2017), and large‐effect alleles that are persisting gene flow could also be located within other genomic regions. Indeed, we found several smaller differentiation peaks on different LGs, suggesting that genomic differentiation between TVEn and TVEf is more widespread across the whole genome than was previously expected due to the sympatric occurrence and connectivity (Barth, Berg, et al., 2017). On the other hand, fixed alleles were not detected. The processes leading to differentiation peaks are complicated and difficult to interpret, and further research including cline analysis, the identification of introgression and the direction and strength of gene flow, identification of selection axes, and analysis of the demographic history (Cruickshank & Hahn, 2014; Noor & Bennett, 2009; Ravinet et al., 2017) will be required to fully determine their importance.

Localization of many weakly differentiated loci across the genome could also be an indication of polygenic adaptation (Pritchard & Di Rienzo, 2010), which would, however, be expected to be quickly broken up through gene flow and recombination if selection is not strong enough, leading to maladapted intermediate genotypes (Lenormand, 2002; Yeaman & Otto, 2011; Yeaman & Whitlock, 2011). Nevertheless, our outlier analyses detected several candidate loci possibly under positive selection, mostly located within regions that have also been identified as differentiation peaks in the genome scans (Supporting Information Figure S4). No significant GO term enrichment was found, but predicted gene models underlying the candidate loci included various genes associated with functions in salinity, temperature and oxygen adaptation: a solute carrier gene (*SLCO1C1*) on LG16 controlling influx and efflux of solutes, which may be important for egg buoyancy regulation under different salinities (Berg et al., 2015); an ion channel (*KCNA10*) on LG01, found to be up‐regulated under salinity stress in blue mussels (Lockwood & Somero, 2011); and the phosphatidylethanolamine *N*‐methyl transferase (*PEMT*) gene located on LG18, which is involved in synthesizing phosphatidylcholine, a major component of membranes shown to occur in different compositions related to salinity and temperature (Athamena et al., 2011; Farkas, Fodor, Kitajka, & Halver, 2001). Two additional genes (*Abhd15* and *PDE3A*, both on LG16) that have previously been associated with temperature adaptation (Dikmen, Cole, Null, & Hansen, 2013; Scott & Johnston, 2012) were also detected. Lastly, a nitric oxide (NO) binding gene (*THAP4*) located on LG08, possibly acting as an NO‐dependent sensor and transcriptional regulator (Bianchetti, Bingman, & Phillips, 2011), was identified. The conversion of nitrite to NO has been shown to occur in fish under hypoxic conditions (Jensen, 2009), thus suggesting a function of *THAP4* in adaptation to low oxygen levels. Interestingly, adaptation to salinity in the Atlantic herring (*Clupea harengus*), a marine fish with similarly high levels of connectivity as Atlantic cod, seems to be immensely polygenic (Lamichhaney et al., 2012) and associated with 10‐ to 200‐kb haplotype blocks that are unlikely to be inversions, but instead predicted to have evolved through the accumulation of multiple causal variants maintained by selection (Martinez‐Barrio et al., 2016). Similarly, in a recently diverged ecotype pair of resident stream and migratory lake sticklebacks that reproduce in sympatry, 19 differentiated regions averaging 267 kb have been suggested to facilitate adaptation (Marques et al., 2016).

Differentiated haplotype blocks may, along with environmentally adaptive alleles, also contain alleles that are associated with prezygotic isolating mechanisms such as behavioural differences, which are important for the reduction of gene flow when postzygotic barriers are incomplete (Kopp et al., 2018). Because different barriers can accumulate during the isolation process and/or act sequentially over the entire life cycle of an organism (Coyne & Orr, 2004; Kulmuni & Westram, 2017), multiple sites may manifest as polygenic traits. In addition, behavioural barriers do not necessarily need to be established as genomic differences, but can also be caused by epigenetic modifications and the subsequent differential expression of genes (i.e., plastic responses), making them more difficult to detect in genome analyses (Ledon‐Rettig, Richards, & Martin, 2013; Reusch, 2014).

### 4.3 Behavioural differences between Atlantic cod types suggest the existence of additional barriers

Behavioural differences that may act as barriers to gene flow include spawning habitat preferences (ecological or spatial isolation, i.e., allopatric reproduction), temporal isolation (e.g., differences in spawning time), and assortative mating. In Atlantic cod, spawning site fidelity (Skjæraasen et al., 2011), natal homing (Bonanomi et al., 2016), and mate choice (Hutchings et al., 1999; Nordeide & Folstad, 2000; Rudolfsen, Figenschou, Folstad, Nordeide, & Soreng, 2005) have been described and discussed to be involved in fitness, local adaptation, and population divergence.

Our main behavioural findings showed that fjord‐type (TVEf) cod utilize generally deeper habitats within the fjord as compared to North Sea‐type (TVEn) cod (Figure 3). Differences in habitat use may lead to a minimization of encounters among the cod types, which could create a prezygotic barrier to gene flow during the spawning season through allopatric reproduction. Contrasting use of different habitat depths is also known from the recently diverged sympatric species pair of *Pundamilia* cichlids (Meier, Marques, Wagner, Excoffier, & Seehausen, 2018; Meier et al., 2017), while in Atlantic cod so far only temporal differences in spawning ground usage among populations have been described (Hüssy, 2011; Hüssy et al., 2016). Interestingly, tagging experiments of two Icelandic Atlantic cod ecotypes showed shared depth usage during the spawning season, but different depth ranges during the feeding period (Pampoulie, Jakobsdóttir, Marteinsdóttir, & Thorsteinsson, 2007). Furthermore, for juvenile Northeast Arctic cod and coastal cod, which co‐occur at northern Norwegian spawning grounds, different settlement depths have been described (Fevolden, Westgaard, Pedersen, & Præbel, 2012). However, the degree to which the differences detected here contribute to reproductive isolation and represent a barrier to gene flow cannot be unequivocally determined due to overlap in space and habitat use. Moreover, limited spatial resolution of acoustic telemetry makes it difficult to locate the individuals at the scale at which reproductive events probably occur. Nevertheless, because cod behaviour is highly variable, both within a single individual (over time) and among individuals (Villegas‐Ríos et al., 2017), the observed differences between cod types are likely grounded on deep ecological differences. Behavioural monitoring at a finer scale (e.g., using a Vemco positioning system, Freitas et al., 2016) during the spawning season will be needed to conclude if the behavioural differences reported in this study translate into actual allopatric reproduction.

Behavioural differences between cod types during the spawning period might also be an indication for assortative mating where alike individuals mate with their kind, leading to reduced gene flow between different ecotypes (Kopp et al., 2018). Significant differences in size between North Sea‐type and fjord‐type cod have been found (Knutsen et al., 2018), generating an opportunity for size‐selective assortative mating (Rueger, Gardiner, & Jones, 2016; Taggart, McLaren, Hay, Webb, & Youngson, 2001). Consistent with this, we found behavioural differences between larger and smaller individuals (Figure 3); however, because individuals were not aged, age‐specific behaviour cannot be excluded. Further investigation through, for example, network statistics (Jacoby & Freeman, 2016) is needed to identify stable interactions between individuals to shed more light on the role of assortative mating in maintaining the cod types.

Nevertheless, assortative mating is also hypothesized to increase the likelihood of producing fit offspring and has been observed in many animals (Jiang, Bolnick, & Kirkpatrick, 2013). Under captive conditions, mate choice and a resulting increase in fitness have also been described for Atlantic cod (Hutchings et al., 1999; Rudolfsen et al., 2005), and male reproductive success was shown to be dependent on the magnitude of the size difference between the female and the male (Bekkevold, Hansen, & Loeschcke, 2002). In our study, fjord‐type (TVEf) cod showed higher survival, indicating higher fitness within the fjord environment as compared to North Sea‐type (TVEn) cod. Such a fitness advantage could arise through natural selection as described above, or through fisheries‐induced selection where Atlantic cod residing at lower depths are more likely to be harvested (Olsen & Moland, 2011; Olsen et al., 2012). However, because the central part of the study area is a no‐take marine reserve where no fishing is allowed, harvesting mortality is unlikely. In a recent paper by Jorde, Synnes et al. (2018), newly spawned eggs of North Sea‐type cod were found inside two fjords, but with a variable pattern between the fjords. One of the fjords remained structured throughout the season with fjord‐type cod dominating inside the fjord, while the other fjord showed fluctuations in cod type frequency, eventually leading to a dominance of fjord‐type cod inside, and North Sea‐type cod outside. This latter observation suggests that fjord‐type cod possibly has a fitness advantage in sheltered fjord habitats, which could, for example, arise through adaptive advantages for oocyte growth and survival in warmer water layers (Bradbury, Snelgrove, & Fraser, 2001; Kjesbu et al., 2010). The Tvedestrand fjord contains several deep basins divided by shallow sills and the water column consists of a surface freshwater layer and saline water underneath where salinity increases with depth, but oxygen decreases (Ciannelli et al., 2010; Halvorsen, 2013). Water temperature is generally more stable and stratified in the fjord as compared to outer coastal or oceanic areas, with deeper layers inside the fjord being comparatively warm in winter and relatively cold in summer (Saetre, 2007). In contrast, exposed outer‐fjord areas may experience extensive mixing that causes the temperature in deeper water to vary more with the seasons (Saetre, 2007). Cod individuals would thus experience less temperature variation inside the fjords than outside. The reduced mean depth of fjord‐type (TVEf) cod indicates residence in water layers with generally lower temperature and oxygen, which could require special adaptations of adults and/or eggs and larvae. In line with this assumption, our outlier analyses detected genes associated with adaptation to temperature and hypoxic conditions (see above). Furthermore, many of the detected genes were also associated with a function in osmoregulation, possibly regulating drift depth of the eggs, where eggs neutrally buoyant in less saline upper layers are retained within the fjord (Ciannelli et al., 2010; Jung et al., 2012). Alternatively, North Sea‐type cod may be better adapted to a life in warmer water layers, while the fjord‐type cod have to seek more stable layers (Freitas et al., 2016). However, adaptation to such habitat has also been discussed in relation to the inversions (see above), indicating that multiple barriers to gene flow may exist.

## 5 CONCLUDING REMARKS

Isolating barriers to gene flow are best studied in differentiated populations that occur in sympatry, where such barriers actively prevent admixture. Here, we demonstrate extensive evidence for the temporally stable sympatric occurrence of genetically and behaviourally differentiated Atlantic cod types within a confined fjord environment. We show that these differences are likely to be maintained through a combination of structural and behavioural barriers to gene flow, both of which may reflect a fitness advantage in local environments. Our study thus emphasizes the high value of genomic analyses for conservation and fisheries management (Bernatchez et al., 2017), while simultaneously highlighting the role of prezygotic, behavioural mechanisms in shaping community structures in the sea of connectivity.

## AUTHOR CONTRIBUTIONS

The study was conceived and designed by J.M.I.B., S.J., E.M.O, and facilitated by S.J. and K.S.J. Genomic data were compiled by B.S. and J.M.I.B. Genomic analyses were performed by J.M.I.B. Behavioural analyses were carried out by C.F., E.M., D.V.‐R. and E.M.O. Samples were provided by C.A., H.K., I.B., J.D., C.P., D.R., J.M. and E.M.O. The manuscript was written by J.M.I.B. with contributions from C.F., D.V.‐R., E.M., E.M.O. and H.K. All authors read and critically revised the manuscript.

## Data Availability

The filtered SNP data set, individual behavioural data, and custom scripts for sliding window analyses are available from the Dryad Digital Repository: https://doi.org/10.5061/dryad.d9c48b6. Whole sequencing read data have been deposited on the European Nucleotide Archive (ENA) under accession number PRJEB29231.
