# Supplementary material for: Disentangling structural genomic and behavioural barriers in a sea of connectivity
Source: Mol Ecol. 2019 Mar 15;28(6):1394–411. doi: 10.1111/mec.15010 (PMC6518941; doi:10.1111/mec.15010)
Supplement: Supplementary file 1 [file MEC-28-1394-s001.pdf]

MOLECULAR ECOLOGY

Supporting Information

Disentangling structural genomic and behavioral barriers in a sea of connectivity

Julia M.I. Barth, David Villegas-Ríos, Carla Freitas, Even Moland, Bastiaan Star, Carl André, Halvor Knutsen, Ian Bradbury, Jan Dierking, Christoph Petereit, David Righton, Julian Metcalfe, Kjetill S. Jakobsen & Esben M. Olsen, Sissel Jentoft

Includes:

Supporting Figures ______ 2

Fig. S1 Hierarchical principal component analysis

Fig. S2 Maximum likelihood model-based ancestry clustering

Fig. S3 Maximum likelihood based phylogenetic inference

Fig. S4 Chromosomal genome scans

Fig. S5 Genome scans of LG16

Fig. S6 Yearly differences in inversion frequency of the Tvedestrand (TVE) sampling site

Fig. S7 Behavioral traits and fate of individual Atlantic cod

Supporting Tables ______ 7

Table S1 Detailed information about Atlantic cod

Table S2 Classification of specimens

Table S3 Absolut allele counts

Table S4 Genes underlying SNPs detected to be under divergent selection

Table S5 GO term enrichment

Supporting Figures

Fig. S1 Hierarchical principal component analysis. Population differentiation was investigated using all samples (Fig. S1a, but see also Fig. 1b), and after exclusion of the most differentiated samples: the eastern Baltic Sea (BOR; Fig. S1b), the North Sea (NOR, LOW; Fig. S1c), and the eastern Baltic and North Sea (Fig. S1d). An analysis focusing solely on the fjord samples (TVE) verified the distribution in two genetically distinct clusters (Fig. S1e), while an analysis including only the TVE individuals grouped within the North Sea cluster (TVEn) together with the North Sea samples (NOR, LOW) verified genomic similarity amongst this group (Fig. S1f). Excluding SNPs with low coverage (DP < 7) yielded qualitatively very similar results (Fig. S1g). For population abbreviations see Table S1.

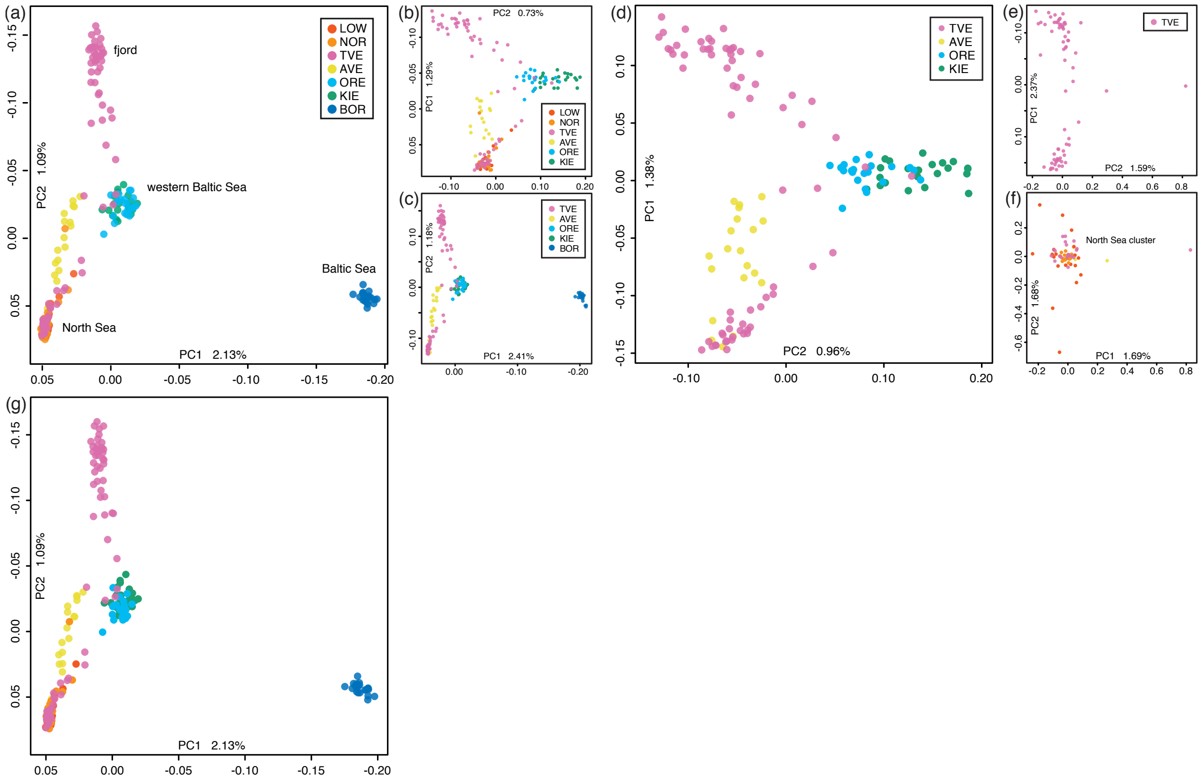

Fig. S2 Maximum likelihood model-based ancestry clustering supported one, two (Fig. S2a), or three (Fig. S2b) cluster according to cross-validation (CV; Fig. S2c). Parameter mean standard error (upper panels) and ancestry proportions (lower panel) are displayed in bars per individual. For population abbreviations see Table S1.

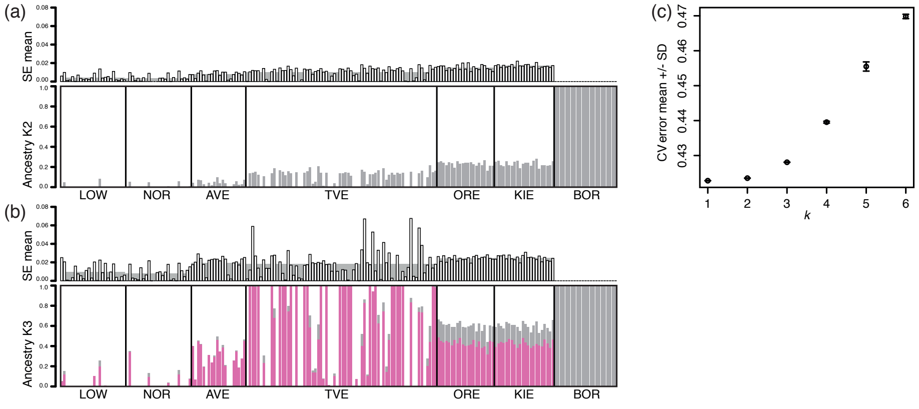

Fig. S3 Maximum likelihood based phylogenetic inference identified three well-supported clades similar to the clustering approaches: the eastern Baltic Sea individuals (BOR) form one monophyletic group, while the fjord specimens (TVE) are found either within a clade including the North Sea specimens (NOR, LOW), or in a separate monophyletic clade, with western Baltic specimens as sister clade. Rooting of the tree with the sister species of Atlantic cod, the Alaskan pollock (*Gadus chalcogrammus*), inferred the western Atlantic Canadian sample as the basal clade to the here included eastern Atlantic population samples. For population abbreviations see Table S1.

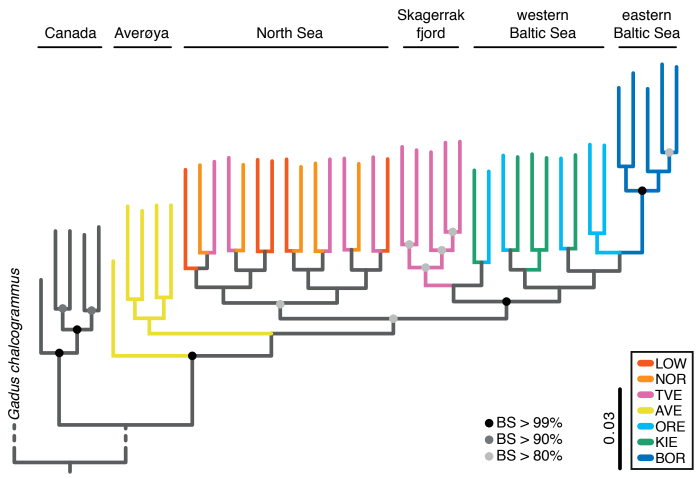

Fig. S4 Chromosomal genome scans in (a) 50, and (b) 100 kb non-overlapping windows, for the following measurements: number of SNPs (# SNPs), linkage disequilibrium (r^2), pairwise fixation index (F_{ST}), pairwise between population sequence divergence (d_{xy};), and nucleotide diversity (π).

See next two pages for Figure.

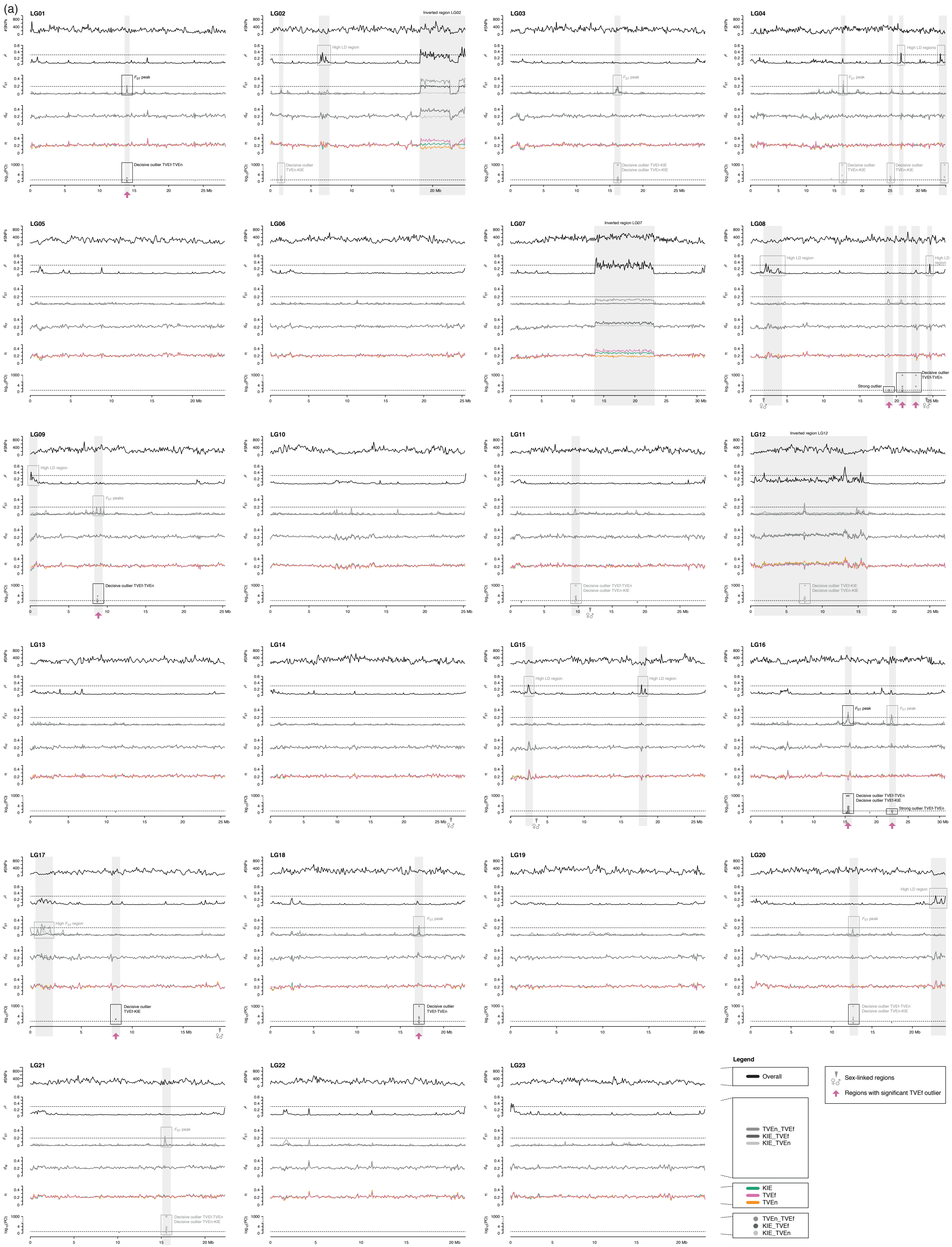
(a)
LG01
LG02
LG03
LG04
LG05
LG06
LG07
LG08
LG09
LG10
LG11
LG12
LG13
LG14
LG15
LG16
LG17
LG18
LG19
LG20
LG21
LG22
LG23
Inverted region LG02
Inverted region LG07
Inverted region LG12
High LD region
High LD regions
Decisive outlier TVEf-TVEn
Strong outlier
Legend
Overall
TVEn_TVEf
KIE_TVEf
KIE_TVEn
KIE
TVEf
TVEn
Sex-linked regions
Regions with significant TVEf outlier

(b)

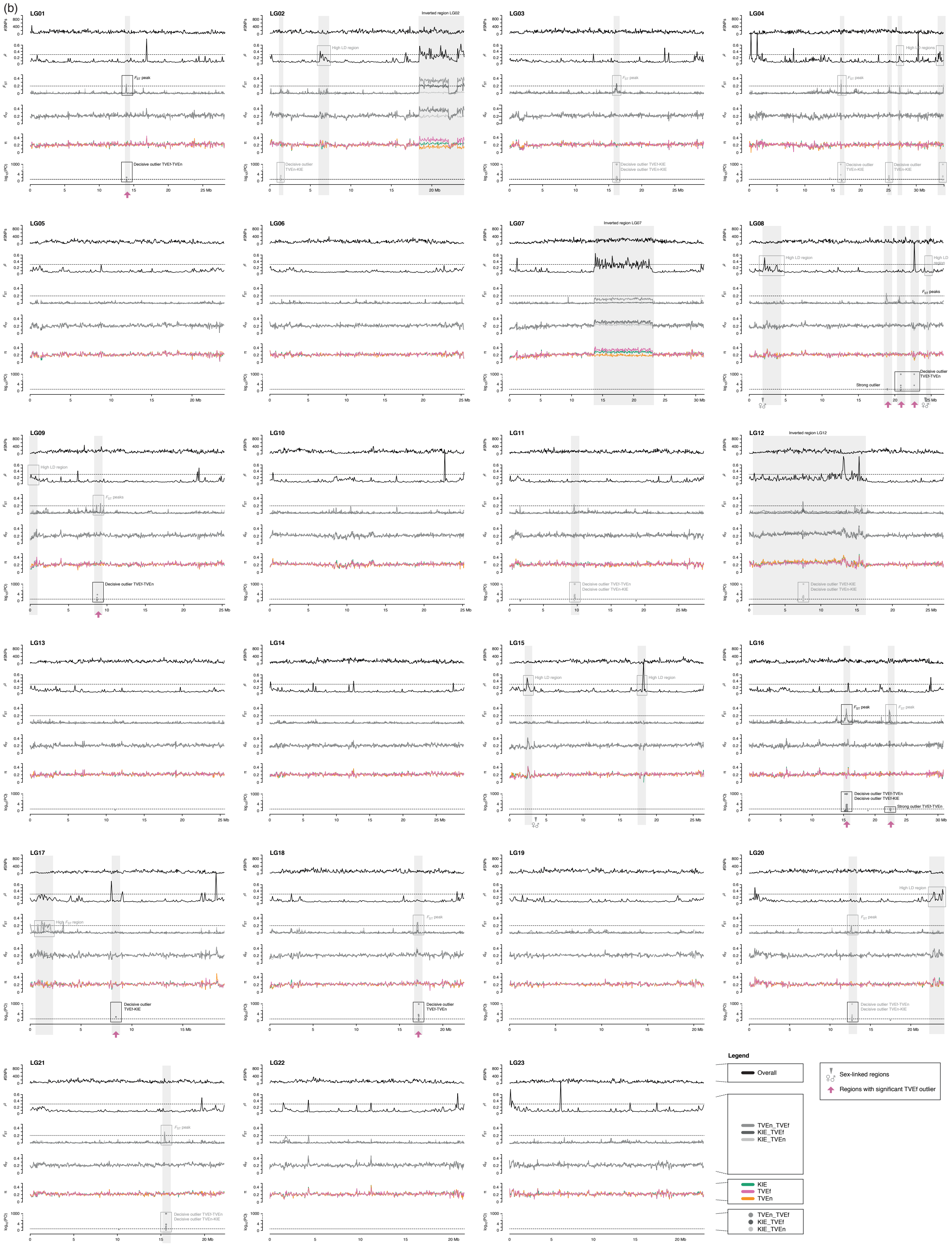
LG01
LG02
Inverted region LG02
LG03
LG04
LG05
LG06
LG07
Inverted region LG07
LG08
LG09
LG10
LG11
LG12
Inverted region LG12
LG13
LG14
LG15
LG16
LG17
LG18
LG19
LG20
LG21
LG22
LG23
Legend
Overall
TVEn_TVEf
KIE_TVEf
KIE_TVEn
KIE
TVEf
TVEn
TVEn_TVEf
KIE_TVEf
KIE_TVEn
Sex-linked regions
Regions with significant TVEf outlier

Fig. S5 Genome scans of LG16 (100 kb non-overlapping windows) for pairwise fixation index (F_{ST}), focusing on the diverged region around position 15,500,000.

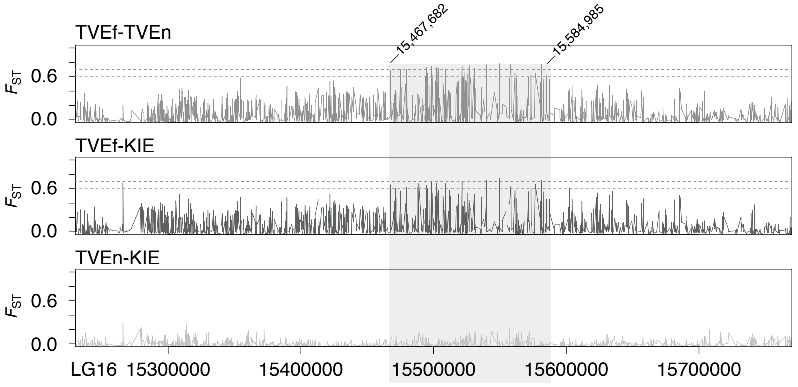

Fig. S6 Yearly differences in inversion frequency of the Tvedestrand (TVE) sampling site. Frequency of homozygous ancestral (white), homozygous inverted (black), and heterozygous (gray) arrangements per sampling year (2011-2013) for the TVE sampling site.

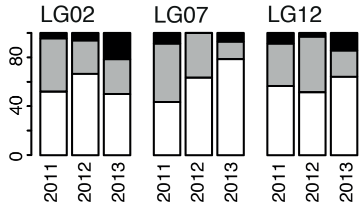

Fig. S7 Behavioral traits and fate of individual Atlantic cod. (a) Raw values of behavioral traits of Atlantic cod monitored by acoustic telemetry in the Tvedestrand fjord, showing monthly home range size (km^2), mean depth use during daytime (m) and diel vertical migration (m) for each individual in the study. (b) Survival of Atlantic cod as time to fate (days) for each individual in the study. TVEf = fjord-type, TVEn = North Sea-type, interm. = intermediate.

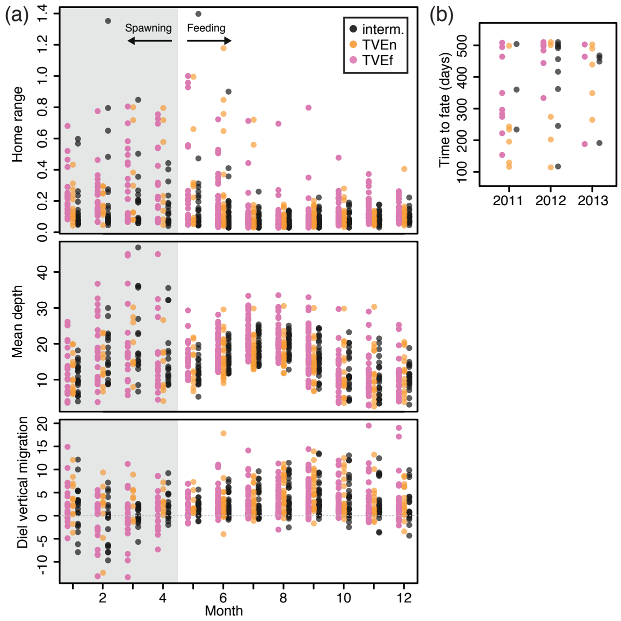

Supporting Tables

Table S1 Detailed information about Atlantic cod (*Gadus morhua*) specimens used in this study. Standard deviation of length and read depth is shown after +/-.

	Sampling	Decimal degrees	Sampling date			Sex	Size	Read depth
**ID**	**Location**	**N, E**	**year**	**month**	**n**	**F:M**	**Length [cm]**	**(coverage)**
AVE	Averøya	63.10, 7.30	2014	September	20	10:10	68.00 +/- 19.63	11.30 +/- 7.29
BOR	Bornholm	ca. 55.21, ca. 15.18	2011, 2012	May	23	11:12	41.65 +/- 10.25	9.15 +/- 1.63
KIE	Kiel Bight	ca. 54.47, ca. 10.42	2011, 2012	February, March	22	10:12	52.41 +/- 12.42	10.86 +/- 2.23
LOW	Lowestoft	52.09, 1.80	2015	March, April	24	17:07	61.08 +/- 9.66	10.47 +/- 0.48
NOR	North Sea	55.57, 5.85	2002	March	24	11:13	65.13 +/- 5.13	8.65 +/- 0.67
ORE	Öresund	ca. 56.01, ca. 12.65	2012	April	21	6:15	53.90 +/- 4.92	8.28 +/- 2.95
TVE	Tvedestand	58.60, 8.95	2011, 2012, 2013	May	70	43:28	45.89 +/- 10.36	10.19 +/- 1.43

Table S2 Classification of specimens according to principal component (PCA) cluster occurrence: eastern Baltic Sea (1), North Sea (2), western Baltic Sea (3), fjord (4), ND/intermediate (5); chromosomal inversions: homozygous ancestral arrangement (0), heterozygous (1), homozygous inverted arrangements (2); and genetic sex according to Star et al. 2016: female (F), male (M). For population abbreviations see Table S1.

	PCA	Inversions			Gen. sex
**ID**	**cluster**	**LG02**	**LG07**	**LG12**	**F/M**
AVE1409002	5	0	1	2	F
AVE1409003	5	1	0	0	M
AVE1409004	5	0	1	1	F
AVE1409005	5	1	0	2	F
AVE1409006	5	0	0	1	M
AVE1409007	2	0	1	0	M
AVE1409008	5	1	0	2	M
AVE1409009	5	0	0	2	F
AVE1409010	5	0	0	2	M
AVE1409012	5	0	2	2	F
AVE1409013	5	1	1	2	F
AVE1409015	5	1	0	2	F
AVE1409016	5	0	0	1	M
AVE1409017	2	0	1	2	M
AVE1409019	2	0	0	1	F
AVE1409020	5	1	1	1	M
AVE1409021	5	1	1	2	F
AVE1409022	5	0	0	2	M
AVE1409023	5	0	0	1	M
AVE1409024	5	1	0	2	F
BOR1104001	1	1	0	1	M
BOR1104002	1	2	1	0	M
BOR1105001	1	1	0	1	F
BOR1105002	1	2	1	0	F
BOR1204001	1	1	0	1	F
BOR1205001	1	2	0	1	M
BOR1205002	1	2	1	2	M
BOR1205003	1	2	0	2	M
BOR1205004	1	1	1	1	M
BOR1205005	1	1	1	0	M
BOR1205006	1	1	0	0	M
BOR1205007	1	2	0	2	M
BOR1205008	1	1	2	1	M
BOR1205009	1	2	1	1	F
BOR1205010	1	2	1	2	F
BOR1205011	1	2	1	0	F
BOR1205012	1	2	0	0	M
BOR1205013	1	1	1	0	F
BOR1205014	1	1	1	0	F
BOR1205016	1	1	1	1	NA
BOR1205017	1	1	0	1	M
BOR1205018	1	2	1	2	F
BOR1205019	1	1	1	0	F
KIE1102002	3	0	0	0	M
KIE1102003	3	1	0	0	M
KIE1102004	3	0	1	1	M
KIE1102005	3	0	2	0	M
NOR020314	2	0	1	1	F
NOR020315	2	0	1	0	F
NOR020316	2	0	0	2	M
NOR020317	2	0	0	2	M
NOR020318	2	0	0	1	F
NOR020319	2	0	0	2	M
NOR020320	5	0	0	0	M
NOR020321	2	0	1	1	M
NOR020322	2	0	0	2	M
NOR020323	2	0	0	1	F
NOR020324	5	0	0	1	F
ORE1203001	3	2	0	0	NA
ORE1203002	3	1	1	0	NA
ORE1203003	3	1	1	1	F
ORE1203004	3	0	0	0	NA
ORE1203005	3	0	0	1	M
ORE1203006	3	0	0	0	M
ORE1203007	3	0	1	1	M
ORE1203008	3	0	0	0	NA
ORE1203009	3	0	0	0	NA
ORE1203010	3	0	0	1	M
ORE1203011	3	0	1	0	NA
ORE1203012	3	1	1	0	M
ORE1203013	3	1	0	0	NA
ORE1203014	3	0	0	0	M
ORE1203015	3	0	1	0	M
ORE1203016	3	0	1	0	NA
ORE1203017	3	1	0	1	M
ORE1203018	3	0	0	1	M
ORE1203019	3	0	0	1	F
ORE1203020	3	0	0	0	M
ORE1203021	3	0	1	0	M
TVE110501	2	0	1	0	F
TVE110502	4	0	0	1	F
TVE110503	4	1	1	0	F
TVE110504	4	0	1	0	M
TVE110505	4	1	0	1	M
TVE110506	2	0	1	2	F
TVE110507	5	1	2	0	F
TVE110508	2	0	0	1	F
TVE110509	2	0	0	0	M
TVE110511	4	0	1	0	F
TVE110512	5	1	0	1	F
TVE110513	2	0	1	2	M
TVE110514	4	1	0	1	F
TVE110515	4	1	2	1	M
TVE110516	4	1	1	0	M

Continued on next page

KIE1102006	3	0	0	0	M
KIE1102007	3	0	2	1	M
KIE1103001	3	1	0	0	F
KIE1103003	3	0	0	0	F
KIE1103004	3	0	1	0	F
KIE1103005	3	0	0	1	F
KIE1103006	3	2	1	1	F
KIE1103007	3	0	1	0	F
KIE1103008	3	0	0	1	F
KIE1103009	3	0	1	0	F
KIE1103013	3	0	0	1	F
KIE1103017	3	0	0	0	M
KIE1103018	3	0	0	0	M
KIE1103019	3	0	0	2	M
KIE1103020	3	0	0	0	M
KIE1203002	3	1	0	1	F
KIE1203003	3	1	1	2	M
KIE1203004	3	0	0	1	M
LOW1503001	2	0	0	0	M
LOW1503002	5	0	1	0	F
LOW1503003	2	0	0	0	F
LOW1503004	2	0	0	0	M
LOW1503005	2	0	0	1	F
LOW1503006	2	0	0	0	F
LOW1503007	2	0	0	0	F
LOW1503008	2	0	0	0	F
LOW1503009	2	0	0	2	F
LOW1503010	2	0	0	1	F
LOW1503011	2	0	0	1	F
LOW1503012	2	0	0	0	F
LOW1503013	5	0	0	0	F
LOW1503014	2	0	0	1	F
LOW1503015	5	0	0	0	F
LOW1504001	2	0	0	0	M
LOW1504002	2	0	0	0	F
LOW1504003	2	0	0	1	M
LOW1504004	2	0	1	0	M
LOW1504005	2	0	0	0	M
LOW1504006	2	0	0	0	M
LOW1504007	2	0	0	0	F
LOW1504008	2	0	0	0	F
LOW1504009	2	0	0	1	F
NOR020301	2	0	0	1	M
NOR020302	5	1	0	0	F
NOR020303	2	0	1	2	M
NOR020304	2	0	0	2	M
NOR020305	2	0	1	1	F
NOR020306	2	0	1	0	M
NOR020307	2	1	1	1	F
NOR020308	2	0	0	2	M
NOR020309	5	0	0	1	F
NOR020310	2	0	0	1	F
NOR020311	2	1	0	2	F
NOR020312	2	0	0	1	M
NOR020313	2	1	0	2	M
TVE110517	5	2	1	0	F
TVE110518	4	1	1	0	M
TVE110519	2	0	0	0	F
TVE110520	4	1	0	0	M
TVE110521	2	0	0	1	F
TVE110522	2	0	0	1	M
TVE110523	4	1	1	0	M
TVE110524	4	0	1	0	F
TVE120501	5	0	0	0	F
TVE120502	5	0	0	1	F
TVE120503	5	0	0	1	F
TVE120504	3	1	0	0	M
TVE120505	4	0	0	1	M
TVE120506	2	0	1	0	M
TVE120507	5	1	1	1	F
TVE120508	2	0	0	2	F
TVE120509	2	0	0	0	F
TVE120510	5	0	0	0	F
TVE120511	2	0	0	1	F
TVE120512	4	2	1	0	F
TVE120513	4	0	0	0	F
TVE120514	4	0	0	0	F
TVE120515	4	2	1	1	M
TVE120516	4	1	1	1	F
TVE120517	2	0	0	1	F
TVE120518	2	0	0	1	F
TVE120519	2	0	0	1	F
TVE120521	3	0	0	0	F
TVE120522	5	1	0	1	M
TVE120523	5	1	0	0	M
TVE120524	4	1	0	1	M
TVE120525	5	1	1	0	F
TVE120526	4	1	1	0	M
TVE120527	5	1	1	0	M
TVE120528	2	0	0	1	M
TVE120529	5	0	1	0	F
TVE120530	5	0	0	1	M
TVE120531	5	0	0	1	F
TVE120532	4	0	1	0	F
TVE120533	4	0	1	0	F
TVE120534	4	0	1	0	F
TVE130501	4	2	0	1	F
TVE130502	4	2	1	1	M
TVE130503	2	0	0	0	F
TVE130504	2	1	0	0	M
TVE130505	5	0	0	0	M
TVE130506	2	0	0	0	F
TVE130507	2	0	0	2	F
TVE130508	5	1	0	0	M
TVE130509	5	1	0	0	M
TVE130510	2	0	0	2	F
TVE130511	5	0	0	0	F
TVE130512	3	0	1	0	M
TVE130513	4	2	2	0	M
TVE130514	4	1	0	1	F

Table S3 Absolut allele counts of the inverted regions for all populations. TVE include all 70 individuals, while TVEf and TVEn are the respective subgroups. For population abbreviations see Table S1.

	**LG02**		**LG07**		**LG12**	
	**Ancestral**	**Inverted**	**Ancestral**	**Inverted**	**Ancestral**	**Inverted**
**AVE**	32	8	31	9	10	30
**BOR**	12	34	31	15	27	19
**KIE**	38	6	34	10	32	12
**LOW**	48	0	46	2	40	8
**NOR**	44	4	41	7	19	29
**ORE**	35	7	34	8	35	7
**TVE**	105	35	109	31	104	36
**TVEf**	32	22	35	19	43	11
**TVEn**	41	1	38	4	24	18

Table S4 Genes underlying SNPs detected to be under divergent selection using Bayesian outlier analysis. Linkage group (LG), gene identifier (ID), gene ontology terms (GO terms).

LG	Start	End	Strand	ID	Name	GO terms
LG01	13972361	13996237	-	GAMO_00024864	Similar to KCNA10: Potassium voltage-gated channel subfamily A member 10 (Gallus gallus)	GO:0005216,GO:0005249,GO:0005515,GO:0006811,GO:0006813,GO:0008076,GO:0016020,GO:0051260,GO:0055085
LG08	18862115	18869555	+	GAMO_00032855	Protein of unknown function	ND
LG08	18865457	18879769	-	GAMO_00032856	Similar to CACNB2: Voltage-dependent L-type calcium channel subunit beta-2 (Oryctolagus cuniculus)	GO:0005245,GO:0005515,GO:0005891,GO:0070588
LG08	20678991	20692859	+	GAMO_00033096	Similar to Thap4: THAP domain-containing protein 4 (Mus musculus)	ND
LG08	20697168	20703674	+	GAMO_00033097	Similar to Rab6b: Ras-related protein Rab-6B (Mus musculus)	GO:0005525,GO:0005622,GO:0006184,GO:0007165,GO:0007264,GO:0015031,GO:0016020
LG08	22523101	22528915	+	GAMO_00033362	Similar to Klrd1: Natural killer cells antigen CD94 (Mus musculus)	GO:0030246
LG08	22531200	22531871	+	GAMO_00033364	Similar to Sft2d3: Vesicle transport protein SFT2C (Mus musculus)	GO:0006810,GO:0016021,GO:0016192
LG09	8644261	8676614	+	GAMO_00044850	Similar to tgl2: Protein-glutamine gamma-glutamyltransferase 2 (Pagrus major)	GO:0003810,GO:0018149
LG09	8676985	8683209	+	GAMO_00044853	Similar to Cyp2j6: Cytochrome P450 2J6 (Mus musculus)	GO:0005506,GO:0016705,GO:0020037,GO:0055114
LG09	8684804	8689784	+	GAMO_00044855	Similar to Sc5d: Lathosterol oxidase (Mus musculus)	GO:0005506,GO:0006633,GO:0016491,GO:0055114
LG16	15260923	15271516	-	GAMO_00009729	Similar to CCDC92: Coiled-coil domain-containing protein 92 (Homo sapiens)	ND
LG16	15412263	15432770	+	GAMO_00009753	Similar to GOSR1: Golgi SNAP receptor complex member 1 (Bos taurus)	GO:0000139,GO:0005801,GO:0006888,GO:0016021
LG16	15491059	15498355	-	GAMO_00009763	Similar to Abhd15: Abhydrolase domain-containing protein 15 (Mus musculus)	ND
LG16	15556186	15567958	+	GAMO_00009770	Similar to PDE3A: cGMP-inhibited 3'%2C5'-cyclic phosphodiesterase A (Homo sapiens)	GO:0003824,GO:0004114,GO:0007165,GO:0008081
LG16	15570798	15572781	+	GAMO_00009771	Similar to PDE3A: cGMP-inhibited 3'%2C5'-cyclic phosphodiesterase A (Homo sapiens)	GO:0004114,GO:0007165
LG16	15605562	15615726	+	GAMO_00009775	Similar to SLCO1C1: Solute carrier organic anion transporter family member 1C1 (Homo sapiens)	GO:0005215,GO:0005515,GO:0006810,GO:0016020
LG16	15623169	15628087	-	GAMO_00009776	Similar to KBTBD3: Kelch repeat and BTB domain-containing protein 3 (Pongo abelii)	GO:0005515
LG16	15631298	15651930	+	GAMO_00009777	Similar to Fam168a: Protein FAM168A (Mus musculus)	ND
LG17	8376662	8378057	+	GAMO_00076508	Similar to ZNF219: Zinc finger protein 219 (Homo sapiens)	GO:0003676,GO:0046872
LG18	17128452	17158062	-	GAMO_00065789	Similar to PEMT: Phosphatidylethanolamine N-methyltransferase (Bos taurus)	GO:0006644,GO:0008170

Table S5 GO term enrichment of genes underlying detected outlier. Uncorrected *p*-value (pval), adjusted *p*-value for multiple tests (padj).

biological process GO:id	Term	Annotated	Significant	Fisher pval	padj
GO:0018149	peptide cross-linking	12	1	0.0170	1.0000
GO:0006888	ER to Golgi vesicle-mediated transport	14	1	0.0200	1.0000
GO:0006633	fatty acid biosynthetic process	17	1	0.0240	1.0000
**Molecular function GO:id**					
GO:0004114	3',5'-cyclic-nucleotide phosphodiesteras...	29	2	0.0004	0.2280
GO:0005506	iron ion binding	121	2	0.0068	1.0000
GO:0003810	protein-glutamine gamma-glutamyltransfer...	9	1	0.0093	1.0000
GO:0005245	voltage-gated calcium channel activity	29	1	0.0298	1.0000
GO:0008170	N-methyltransferase activity	29	1	0.0298	1.0000
**Cellular component GO:id**					
GO:0005801	cis-Golgi network	4	1	0.0041	1.0000
GO:0005891	voltage-gated calcium channel complex	26	1	0.0267	1.0000
GO:0000139	Golgi membrane	44	1	0.0448	1.0000
GO:0008076	voltage-gated potassium channel complex	46	1	0.0468	1.0000
